# The potential of ARL4C and its-mediated genes in atherosclerosis and agent development

**DOI:** 10.3389/fphar.2025.1513340

**Published:** 2025-03-19

**Authors:** Dan Liu, Jie Wang, Shuangshuang Zhang, Hongfei Jiang, Yudong Wu, Chao Wang, Wujun Chen

**Affiliations:** ^1^ Guangdong Provincial People’s Hospital, Zhuhai Hospital (Jinwan Central Hospital of Zhuhai), Zhuhai, Guangdong, China; ^2^ Affiliated Hospital of Qingdao University, Qingdao Cancer Institute, Qingdao University, Qingdao, Shandong, China

**Keywords:** atherosclerosis, cholesterol efflux, ARL4C, ABCA1, agent development

## Abstract

Foam cells are the risk factors for atherosclerosis. Recently, ARL4C, a member of the ADP-ribosylation factor family of GTP-binding proteins, was found to promote cholesterol efflux to decrease foam cell formation, suggesting that ARL4C may be a new promising target for the treatment of atherosclerosis. In fact, ARL4C regulated the expression of multiple atherosis-related genes, including ABCA1, ALDH1A3, ARF6, ENHO, FLNA, LRP6, OSBPL5, Snail2, and SOX2. Many agents, including ABCA1 agonists (CS-6253, IMM-H007, RG7273, and R3R-01), FLNA antagonist sumifilam, LRP6 inhibitor BI-905677 and agonist SZN-1326, and SOX2 inhibitor STEMVAC, were investigated in clinical trials. Targeting these genes could improve the success rate of drug development in clinical trials. Indeed, many agents could regulate ARL4C expression, including LXR/RXR agonists, Ac-LDL, sucrose, T9-t11-CLA, and miR-26. Downregulation of ARL4C with siRNA and anti-sense oligonucleotide (ASO), such as ASO-1316, is developing in preclinical research for the treatment of lung adenocarcinoma, liver cancer, and colorectal cancer. Thus, ARL4C and its regulated genes may be a potential target for drug development. Thus, we focus on the role of ARL4C and its-mediated genes in atherosclerosis and agent development, which provide insights for the identification, research, and drug development of novel targets.

## 1 Introduction

**FIGURE 1:**
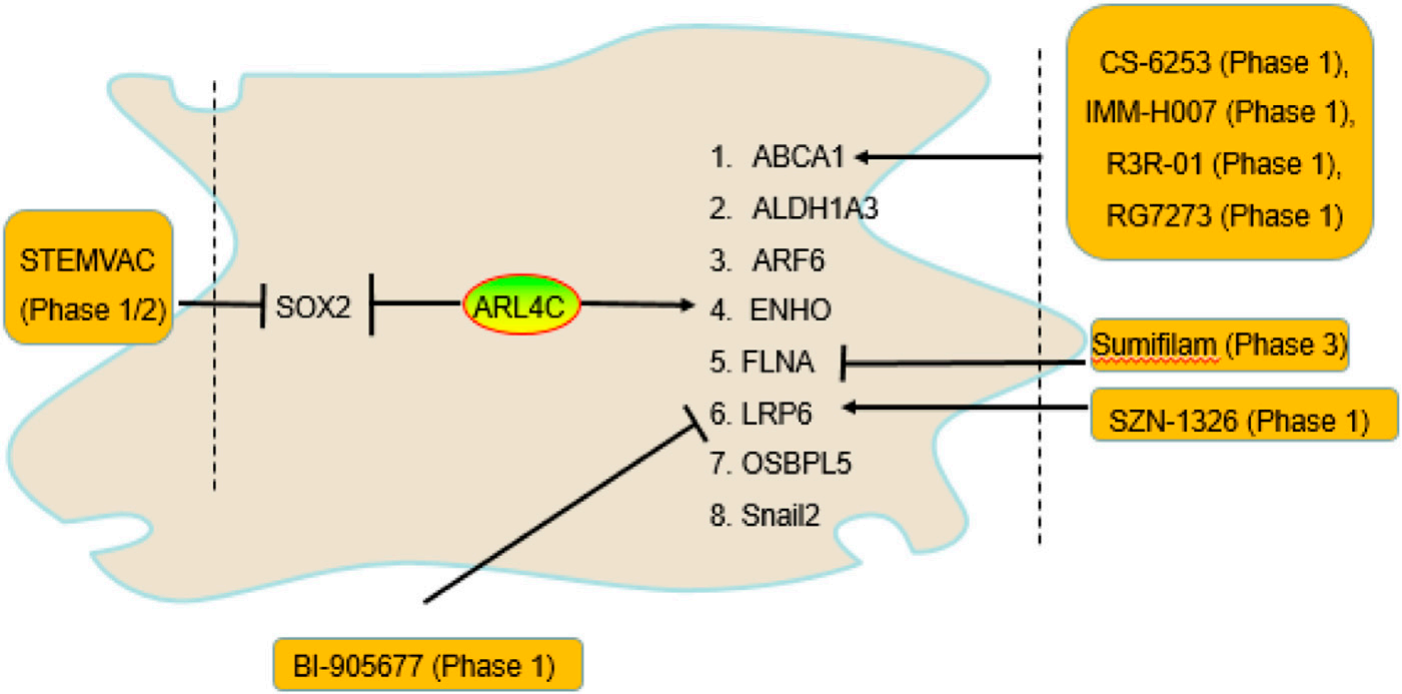
The genes regulated by ARL4C and drugs that entered clinical trials. ARL4C promoted ABCA1, ALDH1A3, ARF6, ENHO, FLNA, LRP6, OSBPL5, and Snail2 expression and suppressed SOX2 expression. Many agents targeting ABCA1, FLNA, LRP6, and SOX2, including BI-905677, CS-6253, IMM-H007, R3R-01, RG7273, sumifilam, SZN-1326, and STEMVAC, have entered clinical trials.

ADP-ribosylation factor-like 4C (ARL4C, also known as ARF-like 7 (ARL7)), a member of the ADP-ribosylation factor family of GTP-binding proteins, was first discovered from a lymphokine-activated T-killer (TLAK) cell subtraction library. ARL4C plays a key role in microtubule dynamics and cell morphology changes (Fujii et al., 2022; Zhang et al., 2022). Recently, a research reported that the ARL4C promotes vesicular cholesterol trafficking to the plasma membrane to enhance cholesterol efflux from intracellular pools to ATP-binding cassette transporter A1 (ABCA1), ABCG1, and apoA-I (El Roz et al., 2013). The promoter of ARL4C has a liver X-receptor (LXR) response element (LXRE) sequence and may be an integral part of LXR-dependent cholesterol efflux, suggesting that ARL4C is a direct target gene of LXRs (LXRα and LXRβ). Knockdown of ARL4C also regulates genes that are involved in cholesterol metabolism and atherosclerosis with GO enrichment analysis (Yang et al., 2022), suggesting that ARL4C regulates atherosclerosis development. In fact, ARL4C can regulate the expression of multiple atherosclerosis-related genes (Figure 1), including ABCA1, aldehyde dehydrogenase 1 family member A3 (ALDH1A3), ARF6, energy homeostasis associated (ENHO), filamin-A (FLNA), low-density lipoprotein receptor-related protein-6 (LRP6), oxysterol binding protein like 5 (OSBPL5, also named ORP5), and snail family zinc finger 2 (Snail2, also named SLUG), and sex-determining region Y-box 2 (SOX2) expression (Yang et al., 2022; Chen et al., 2021a; Hofmann et al., 2007; Chiang et al., 2017; Hu et al., 2018; Wang et al., 2018). However, whether these genes are pro-atherosclerotic or anti-atherosclerotic depends on their location, suggesting that the role of ARL4C in atherosclerosis may depend on its location. Notably, many agents that target ARL4C-mediated genes, including ABCA1, FLNA, LRP6, and SOX2, have been approved for clinical trials, which suggests that targeting these genes could greatly improve the success rate of drug development. This review focused on the potential of ARL4C and its-mediated genes in atherosclerosis and agent development in the hope of providing knowledge for identifying drug development targets.

## 2 The potential role and mechanism of ARL4C in cholesterol efflux

Vesicular transport, such as that of giant plasma membrane vesicles (GPMVs), is an important form of intracellular cholesterol transport. GPMVs are rich in free cholesterol to facilitate cholesterol efflux from cell membranes (Sedgwick et al., 2018; Wei et al., 2009). GPMV formation requires microtubules and the actin cytoskeleton but does not require vimentin or keratin 17. Microtubule marker α-Tubulin promotes GVMP formation and cholesterol efflux by regulating the anchorage sites of microtubules (Sedgwick et al., 2018; Wei et al., 2009). Interestingly, ARL4C increased GVMP formation and vesicular transport by interacting with α-tubulin in a GTP- or GDP-independent binding state and promoting actin remodeling and polymerization, suggesting that ARL4C promoted cholesterol efflux by promoting GVMP formation through the enhancement of microtubules and the actin cytoskeleton (El Roz et al., 2013; Sedgwick et al., 2018; Wei et al., 2009). In fact, ARL4C is mainly localized in the cell membrane and cytoplasmic vesicles and is characterized by rapid nucleotide exchange and nuclear localization signals (Wei et al., 2009). The stretches of basic amino acids of the C terminus of ARL4C protein is a nuclear localization signal. The shuttle of ARL4C between the nucleus and intracellular organelles depends on its GTP/GDP-binding status (Jacobs et al., 1999). ARL4C is rapidly recruited to cytoplasmic vesicles in a manner dependent on its myristoylation when intracellular cholesterol is excessive and then promotes vesicle formation and transport to enhance cholesterol efflux.

**FIGURE 2:**
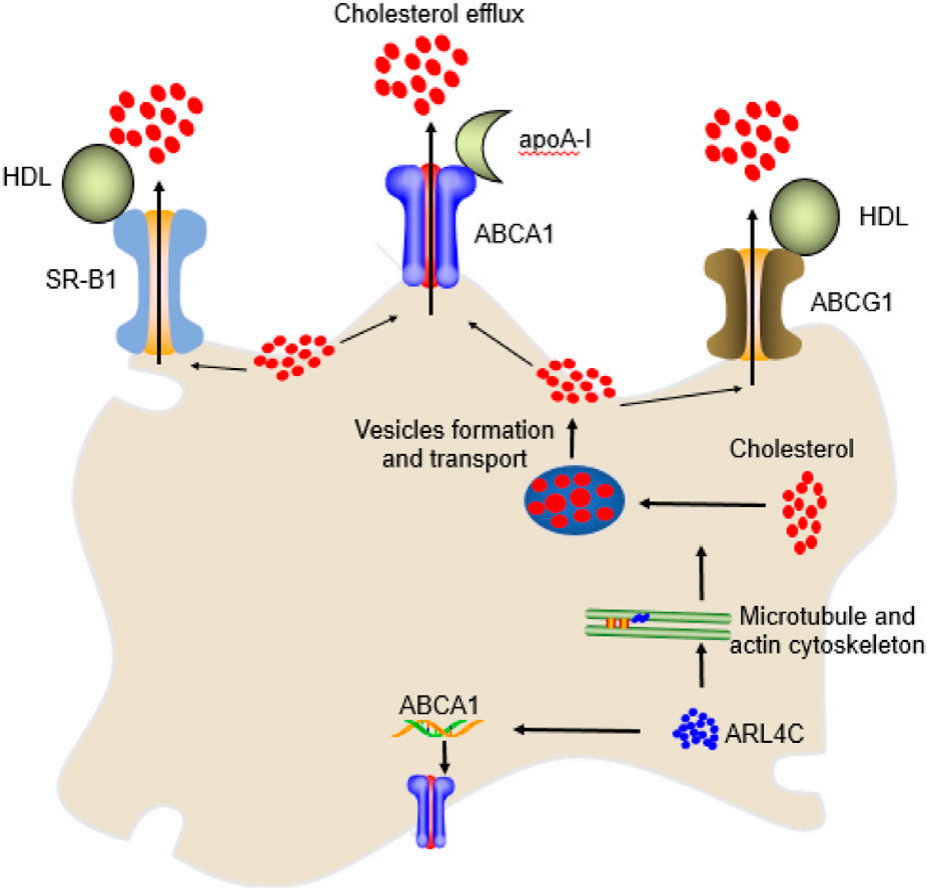
The mechanism of ARL4C in cholesterol efflux. ARL4C promoted the transport of cholesterol from the perinuclear compartment to the plasma membrane for ABCA1, ABCG1, and SR-B1 to be transported extracellularly by promoting microtubules and the actin cytoskeleton and their mediated vesicle formation and transport. ARL4C also promoted ABCA1 expression to enhance cholesterol efflux.

Many studies have shown that ARL4C promotes cholesterol efflux to apoA-I (El Roz et al., 2013; Sedgwick et al., 2018; Wei et al., 2009). Overexpression of ARL4C by 3-fold enhanced apoA-I-mediated cholesterol efflux by 2.8-fold in HeLa cells, whereas downregulation of ARL4C by 3-fold reduced cholesterol efflux by 0.5-fold. ARL4C knockdown increased total and free cholesterol but not cholesterol esters in cells, suggesting that ARL4C does not regulate cholesterol esters. Notably, overexpression of ABCA1 only weakly suppressed the ability of ARL4C shRNA to increase cholesterol levels (Yang et al., 2022), suggesting that ARL4C promotes cholesterol efflux partially through ABCA1. ARL4C knockdown suppressed not only apoA-I-mediated cholesterol efflux but also HDL. The macrophage-specific ARL4C mutation (mutation ARL4C promoter LXRE sequences) also promoted foam cells and reduced reverse cholesterol transport (RCT, a process that transfers cholesterol from peripheral cells to the liver through the blood circulation for metabolic transformation and excretion) in LDLR^−/−^ mice (Table 1) (Yin et al., 2020). Knockdown of ARL4C with shRNA decreased ABCA1 expression (Yang et al., 2022). As mentioned above, cholesterol efflux to HDL is mainly controlled by ABCG1 and SR-B1, suggesting that ARL4C promotes cholesterol efflux by promoting intracellular cholesterol transport to ABCA1, ABCG1, and SR-B1 and/or enhancing ABCA1 expression (Figure 2). In addition, endogenous apoA-I promoted GVMP formation and the accumulation of GPMVs on the PM by enhancing actin polymerization. We found that apoA-I is expressed not only in hepatocytes and enterocytes but also in monocyte-macrophages, dendritic cells (DCs), and T cells, suggesting that ARL4C works with apoA-I to stimulate vesicle formation, transport, and cholesterol efflux.

**TABLE 1:** The role of ARL4C in atherosclerosis.

Cell/aminal	Function	References
MCF-7 breast cancer cells	t9,t11-CLA increased cholesterol efflux and suppressed cell proliferation by enhancing ARL4C expression	El Roz et al. (2013)
HeLa cells	ARL4C promotes cholesterol efflux	Sedgwick et al. (2018)
LDLR^−/−^ mice	Macrophage-specific ARL4C mutation promoted foam cells	Yin et al. (2020)
HeLa cells	LXR agonist T0901317and RXR agonist RO-26-4456 increased cholesterol efflux by enhancing ARL4C expression	Sun et al. (2012)
RAW264.7 cells, THP-1 cells	LXR agonist T0901317 increased cholesterol efflux by enhancing ARL4C expression	Sun et al. (2012)
RAW264.7 cells, THP-1 cells, and HepG2 cells	MiR-26 suppressed cholesterol efflux by targeting ARL4C	Sun et al. (2012)
RAW264.7 cells, THP-1 cells, human peripheral blood-derived monocytes, WT macrophages, LXRα^−/−^ macrophages, and LXRβ^−/−^ macrophages	LXR agonist GW3965 or T01317 and RXR LG268 agonist increased ARL4C expression	Hong et al. (2011)
LXRα/β−/− macrophages	LXR agonist GW3965 or T01317 and RXR LG268 agonist did not change ARL4C expression	Hong et al. (2011)
C57Bl/6 mice	LXR agonist GW3965 increased ARL4C expression in the liver and spleens	Hong et al. (2011)

## 3 The potential role and mechanism of the ARL4C-mediated gene in atherosclerosis

### 3.1 ABCA1

**FIGURE 3:**
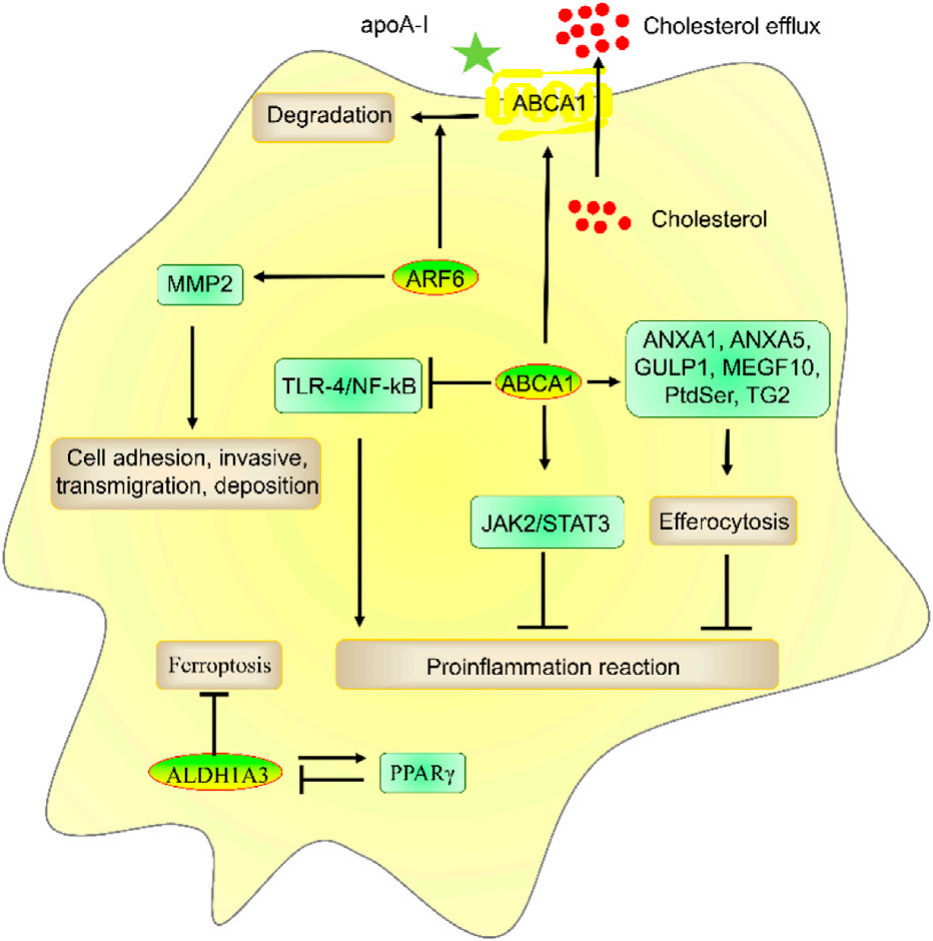
The role and mechanism of ABCA1, ALDH1A3, and ARF6 in atherosclerosis risk factors. ABCA1 promoted apoA-I-mediated cholesterol efflux and ANXA1-, ANXA5-, GULP1-, MEGF10-, PtdSer-, and TG2-mediated efferocytosis and suppressed TLR-4/NF-kB- and JAK2/STAT3-mediated inflammatory reactions. ALDH1A3 suppressed ferroptosis and formed a negative feedback loop with PPARγ, which plays a key role in preventing atherosclerosis. ARF6 reduced cholesterol efflux by promoting ABCA1 degradation and promoted invasive capacities by enhancing MMP2 activation.

As noted above, ABCA1 promotes cholesterol efflux by binding to apoA-I. Previous studies from our and others’ laboratories have shown that ABCA1 promotes RCT to suppress foam cell and atherosclerotic plaque formation (Chen et al., 2020b; Chen et al., 2021b; Chen et al., 2020a; Chen et al., 2021c; Chen et al., 2021d; Chen et al., 2021e; Zhang et al., 2021b). Many studies have shown that ABCA1 also decreases proinflammatory reactions by reducing the toll-like receptor-4 (TLR-4)/nuclear factor kB (NF-kB) proinflammatory pathway and enhancing the JAK2/STAT3 anti-inflammatory pathway (Bi et al., 2015; Matsuo, 2022; Wang et al., 2022a). In addition, ABCA1 increases efferocytosis, which is an apoptotic cell and inflammatory factor clearance process, by regulating the expression of annexin A1 (ANXA1), ANXA5, engulfment adaptor phosphotyrosine-binding domain (PTB) domain containing 1 (GULP1), multiple EGF-like domains 10 (MEGF10), phosphatidylserine (PtdSer), and transglutaminase 2 (TG2) (Figure 3) (Chen et al., 2021c; Chen et al., 2021b). Thus, ABCA1 plays an important role in reducing atherosclerosis development.

### 3.2 ALDH1A3

ALDH1A3 can convert acetaldehyde to acetate to produce acetyl-CoA, pyruvate, and citrate (Li et al., 2021). ALDH1A3 suppressed ferroptosis (Kram et al., 2022; Hua et al., 2018). ALDH1A3 also increased PPARγ expression. However, PPARγ decreased ALDH1A3 expression, which suggests that ALDH1A3 and PPARγ form a negative feedback loop (Hua et al., 2018). PPARγ plays a key role in inhibiting atherosclerosis (Szanto et al., 2021), which suggests that ALDH1A3 is related to atherosclerosis by suppressing ferroptosis and enhancing PPARγ expression. However, the role of ALDH1A3 in atherosclerosis is unclear. In addition, the potential of ALDH1A3 as a target for disease diagnosis and drug development has not been investigated. More studies are needed.

### 3.3 ARF6

ARL4C activates ARF6 by recruiting cytohesins to the plasma membrane (Hofmann et al., 2007; Han et al., 2020). Many studies have shown that ARF6 plays a key role in atherosclerosis. For example, ARF6 promoted VSMC invasive capacities by stimulating matrix metalloproteinase-2 (MMP2) and MMP14 activation (Fiola-Masson et al., 2022). ARF6 promoted vascular oxidative stress and endothelial dysfunction in ECs and reduced cholesterol efflux by promoting ABCA1 degradation in RAW264.7 cells (Wanschel et al., 2021; Mukhamedova et al., 2016). However, the pathogenesis of atherosclerosis is very complex. The role of ARF6 in atherosclerosis *in vivo* is unclear. We cannot determine the atherogenic effect of ARF6 based on the *in vitro* results alone. In addition, cholesterol efflux is mainly controlled by ARF6-independent pathways (Mukhamedova et al., 2016). As mentioned above, ARL4C promoted apoA-I-mediated cholesterol efflux by promoting intracellular cholesterol transport and ABCA1 expression, which suggests that ARL4C-mediated degradation of ABCA1 is not sufficient to weaken ARL4C-mediated ABCA1 expression and cholesterol efflux.

### 3.4 ENHO

**FIGURE 4:**
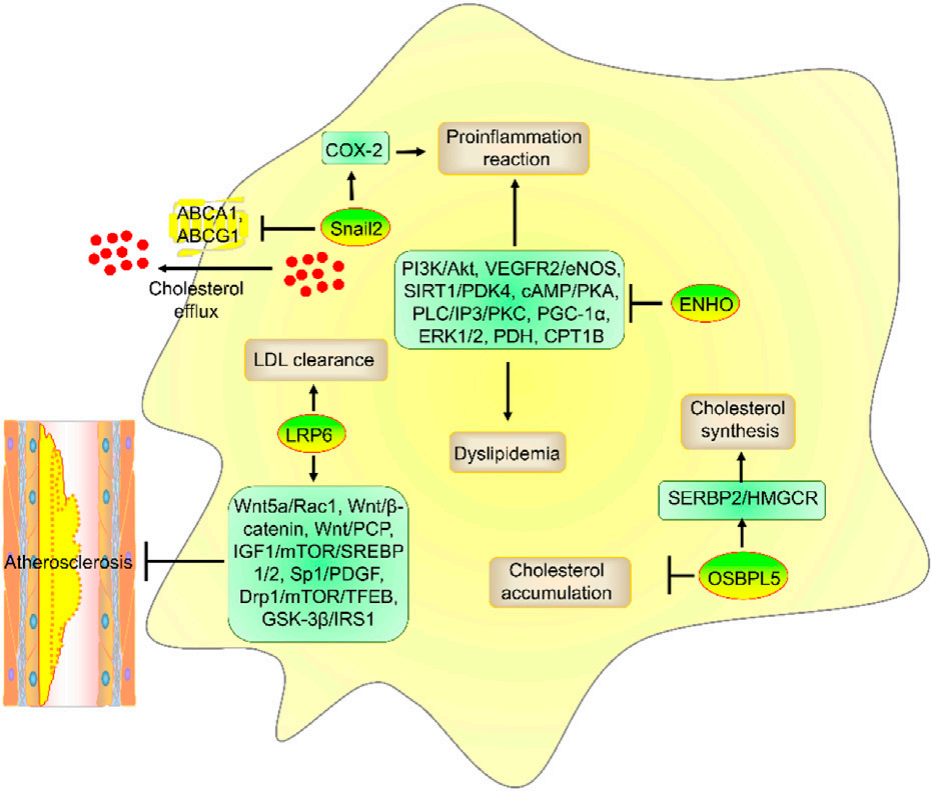
The role and mechanism of ENHO, LRP6, OSBPL5, and Snail2 in atherosclerosis risk factors. ENHO suppressed dyslipidemia and proinflammatory reactions by suppressing PI3K/Akt, VEGFR2/eNOS, ERK1/2, PDH, SIRT1/PDK4, cAMP/PKA, PLC/IP3/PKC, PGC-1α, and CPT1B. LRP6 promoted LDL clearance. OSBPL5 promoted cholesterol synthesis by enhancing the SREBP2/HMGCR axis but decreased cholesterol accumulation. Moreover, Snail2 promoted proinflammatory reactions by enhancing COX-2 expression and suppressed cholesterol efflux by reducing ABCA1 and ABCG1 expression.

ENHO encodes adropin protein and is suppressed by liver X-receptors (LXRs) (Niepolski and Grzegorzewska, 2016). ENHO is a biomarker in obesity and dyslipidemia (Muhammed et al., 2022). Many studies have shown that adropin suppresses dyslipidemia and atherosclerosis progression by regulating PI3K/Akt, vascular endothelial growth factor receptor-2 (VEGFR2)/endothelial nitric oxide synthase (eNOS), ERK1/2, pyruvate dehydrogenase (PDH), silent information regulator sirtuin 1 (SIRT1)/pyruvate dehydrogenase kinase 4 (PDK4), cAMP/PKA, PLC/IP3/PKC, peroxisome proliferator-activated receptor-γ coactivator-1α (PGC-1α), and carnitine palmitoyltransferase 1B (CPT1B) (Figure 4) (Niepolski and Grzegorzewska, 2016; Jaiswal et al., 2021; Li et al., 2016; Mocnik and Marcun Varda, 2022), which suggests that ARL4C suppresses dyslipidemia and atherosclerosis progression by enhancing ENHO expression.

### 3.5 FLNA

**FIGURE 5:**
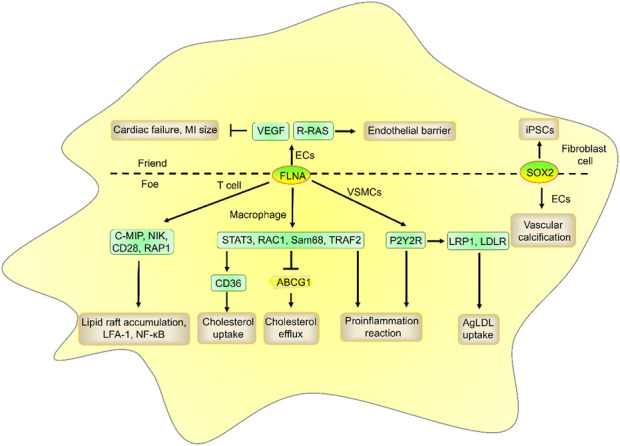
The role and mechanism of FLNA and SOX2 in atherosclerosis risk factors. FLNA from macrophages, VSMCs, and T cells promoted cholesterol uptake, proinflammatory reactions, and lipid accumulation and suppressed cholesterol efflux, while endothelial FLNA suppressed cardiac failure and MI size and increased endothelial barrier function. Fibroblast SOX2 reduced proinflammatory responses by inducing iPSCs, while endothelial SOX2 promoted vascular calcification.

ARL4C promoted filopodium formation and cell migration activation by enhancing the faciogenital dysplasia 6 (FGD6)/cell division cycle 42 (CDC42) pathway by binding and interacting with FLNA in HeLa and A549 cells (Chiang et al., 2017). Cell migration is a crucial step in wound healing and remodeling in MI and atherosclerosis. FLNA is a large actin-binding cytoskeleton protein that plays an important role in cell movement (Zhou et al., 2021). Mutation or lack of FLNA induces cardiovascular malformations, such as heart and vessel anomalies, in humans. However, the role of FLNA in atherosclerosis depends on its location. Specifically, VSMC FLNA promoted cell migration, proinflammatory cytokine lymphotoxin-α (LTA) secretion, and LRP1 and LDLR-mediated aggregated LDL (agLDL) uptake by binding and interacting with the purinergic receptor P2Y2 (P2Y2R) (Dissmore et al., 2016). Macrophage FLNA enhances CD36-mediated cholesterol uptake, cell migration and proliferation and NF-κB-mediated proinflammatory cytokine secretion (such as IL-1β, IL-6, IL-12, and TNF-α secretion) and suppresses ABCG1-mediated cholesterol efflux by interacting with signal transducer and activator of transcription 3 (STAT3), RAS-related C3 botulinum toxin substrate 1 (RAC1), Src-associated-in-mitosis-68-kDa (Sam68), and TNFR-associated factor 2 (TRAF2) (Sharma et al., 2020; Han et al., 2019; Bandaru et al., 2020). T-cell FLNA promoted lipid raft accumulation, LFA-1 expression, and NF-κB-mediated proinflammatory cytokine secretion by interacting with C-MIP, NF-kB-activating kinase (NIK), CD28, and RAP1 (Grimbert et al., 2004; Tavano et al., 2006). However, endothelial FLNA suppressed cardiac failure and the size of the MI by enhancing VEGF expression and VEGF-mediated angiogenesis (Bandaru et al., 2015). Endothelial FLNA also increases the function of the endothelial barrier by interacting with R-RAS (Griffiths et al., 2011). These results suggest that FLNA from macrophages, VSMCs, and T cells may exhibit proatherogenic effects, whereas endothelial FLNA may exhibit antiatherogenic effects (Figure 5).

### 3.6 LRP6

LRP6 is a member of the LDLR superfamily and plays a key role in LDL clearance. Downregulation of LRP6 activity promotes multiple risk factors for atherosclerosis, including decreased serum LDL, glucose, and triglyceride levels (Desita et al., 2022). LRP6 suppressed VSMC differentiation and atherosclerosis by suppressing platelet-derived growth factor (PDGF) expression. Clinical and genomic trials have also shown that LRP6 genetic variants promote atherosclerosis development (Escate et al., 2017; Kumar et al., 2022; Rajamannan, 2011). Indeed, LRP6 can inhibit the development of atherosclerosis by regulating several signaling pathways, including the Wnt5a/Rac1, Wnt/β-catenin, Wnt/PCP, Sp1-dependent PDGF, glycogen synthase kinase 3 beta (GSK-3β)/insulin receptor substrate 1 (IRS1), insulin-like growth factor 1 (IGF1)/mechanistic target of rapamycin kinase (mTOR)/sterol response element binding protein 1/2 (SREBP1/2), and dynamin-related protein 1 (DRP1)/mTOR/transcription factor EB (TFEB) signaling pathways (Alrefaei and Abu-Elmagd, 2022; Kang, 2020). Interestingly, ARL4C promoted the expression of LRP6, WNT5A, and WNT11 (Guo et al., 2018), which suggests that ARL4C suppresses atherosclerosis development by enhancing LRP6 expression. However, the mechanism by which ARL4C affects LRP6 expression is unclear.

### 3.7 OSBPL5

OSBPL5 is a member of the OSBP family. OSBPL5 promoted autophagy and intracellular cholesterol transport from late endosomes/lysosomes (LEs/LYs) to the ER and cell membrane. OSBPL5 depletion promoted cholesterol accumulation in LEs/LYs and subsequently induced foam cell formation and atherosclerosis development (Santos et al., 2020; Yu et al., 2014). Interestingly, knockdown of ARL4C with shRNA reduced cholesterol transport from LEs/LYs and autophagy by decreasing OSBPL5 expression *via* the Notch-RBP-Jκ-histone 3 lysine 4 trimethylation (H3K4Me3) pathway (Yang et al., 2022), which suggested that ARL4C suppressed cholesterol accumulation and foam cell formation by enhancing OSBPL5 expression. Notably, OSBPL5 also promotes SREBP2 expression to induce the downstream gene HMG-CoA reductase (HMGCR), which is the rate-limiting enzyme in cholesterol synthesis (Ishikawa et al., 2010; Santos et al., 2020), suggesting that ARL4C may promote HMGCR expression and cholesterol synthesis. However, the knockdown of ARL4C or OSBPL5 promoted cholesterol accumulation, which suggested that ARL4C- or OSBPL5-mediated HMGCR expression was not sufficient to increase intracellular cholesterol levels.

### 3.8 Snail2

ARL4C knockdown reduced Snail2 expression in AGS and 58As9 cells (Hu et al., 2018). However, the mechanism by which ARL4C affects Snail2 is unclear. Snail2 promoted epithelial-to-mesenchymal transition (EMT) and endothelial-to-mesenchymal transition (EndMT). Snail2 promoted atherosclerosis development by enhancing the transformation of VSMCs toward an inflammatory phenotype by activating the cyclooxygenase-2 (COX-2)/prostaglandin E2 (PGE2) pathway and suppressing cholesterol efflux by reducing ABCA1 and ABCG1 promoter expression in VSMCs (Yuan et al., 2022; Ledard et al., 2020). Thus, ARL4C may promote atherosclerosis development by enhancing Snail2 expression. However, the Snail2/ABCA1 and ABCG1 axes did not change ARL4C-mediated cholesterol efflux.

### 3.9 SOX2

ARL4C promoted SOX2 expression in glioblastoma (GBM) cells (Chen et al., 2021a). SOX2 is a stem cell and mesenchymal marker. Endothelial-specific deletion of SOX2 reduces vascular calcification to decrease atherosclerotic plaque burden in apoE^−/−^ mice (Bostrom et al., 2016; Zhang et al., 2021a), which suggests that endothelial SOX2 may be a proatherogenic gene. However, SOX2 can successfully program adult human fibroblasts into human induced pluripotent stem cells (iPSCs), which reduce proinflammatory responses and atherosclerosis development by decreasing TNFα and IL-6 levels (Wong et al., 2013; Toyohara et al., 2020; Shi et al., 2018). Many studies have shown that human iPSCs are promising therapies for the treatment of cardiovascular diseases (Karimian et al., 2023; Mahmud et al., 2022; Mansfield et al., 2022). Thus, SOX2 exhibits both atheroprotective and proatherogenic effects. However, additional studies are needed to evaluate whether the regulation of SOX2 by ARL4C is pro-atherosclerotic or anti-atherosclerotic.

Taken together, the downstream genes of ARL4C, including ABCA1, ALDH1A3, ARF6, ENHO, FLNA, LRP6, OSBPL5, Snail2, and SOX2 play an important role in atherosclerosis. Thus, we hypothesize that ARL4C may regulate atherosclerosis development by regulating these downstream genes. Notably, the role of these downstream genes (except ABCA1) in atherosclerosis is not direct but indirectly affects atherosclerosis by regulating other genes or proteins. More studies are needed to confirm this hypothesis.

## 4 Agent development of targeting the ALR4C-mediated genes ABCA1, FLNA, LRP6, and SOX2

As mentioned above, ARL4C included multiple downstream genes, including ABCA1, ALDH1A3, ARF6, ENHO, FLNA, LRP6, OSBPL5, Snail2 and SOX2. We searched for agents that target these genes with Adisinsight, Bing, Chinadrugtrials, ClinicalTrials, Glgoo, Pharnexcloud, PubChem Compound, Pubmed, and Zhihuiya. However, in our power, we only found 4 genes agents, including ABCA1, FLNA, LRP6, and SOX2, which were investigated in clinical trials. Thus, ABCA1, FLNA, LRP6, and SOX2 are promising targets for drug development.

### 4.1 ABCA1

#### 4.1.1 CS-6253

CS-6253 (also named CS6253 and Cogpep) is an alpha-helical peptide designed from the C-terminus of apoE that serves as an ABCA1 agonist. The use of CS-6253 in preclinical trials for the treatment of Alzheimer’s disease (AD), atherosclerosis, and type 2 diabetes (T2DM) is being developed (Hafiane et al., 2019; Noveir et al., 2022; AdisInsight, 2022). The development of CS-6253 was supported by SBIR grants from the National Institutes of Health and the National Institute of Aging for the initiation of first-in-human trials on 22 November 2021 (AdisInsight, 2022). Early phase 1 of CS-6253 for the treatment of AD was also initiated on 28 July 2023 (NCT05965414). However, to our knowledge, the role of CS-6253 in atherosclerosis has not been investigated in clinical trials. No specific ABCA1 agonists have entered phase 2 clinical trials. Additional studies are needed to confirm the feasibility of using ABCA1 as a target for drug development.

#### 4.1.2 IMM-H007

Triacetyl-3-hydroxyphenyladenosine (IMM-H007, also named H007, THPA, and WS070117), a derivative of cordycepin, was investigated in phase 1 clinical trial for the treatment of dyslipidemia on 16 March 2022 (CTR20220514). IMM-H007 reduced atherosclerosis development by suppressing ABCA1 degradation in preclinical trials (Huang et al., 2015). However, IMM-H007 is not a specific ABCA1 agonist. IMM-H007 is also an AMP-activated protein kinase (AMPK) agonist and transforming growth factor β1 (TGFβ1) antagonist (Gao et al., 2019). IMM-H007 decreased TNFα, IL-1, IL-6, malondialdehyde (MDA), monocyte chemoattractant protein 1 (MCP-1), inducible nitric oxide (NO) synthase (iNOS), lectin-like oxidized LDL receptor-1 (LOX-1), and myeloperoxidase (MPO) expression and increased ABCG1, Akt, apoA-I, arginase 1 (Arg-1), eNOS, IL-10, lecithin-cholesterol acyltransferase (LCAT), NO, phosphorylated AMPK (pAMPK), paraoxonase 1 (PON1), and SR-B1 expression in mice, suggesting that IMM-H007 not only promoted cholesterol efflux and endothelial protection but also suppressed proinflammatory reactions and cholesterol uptake (Zhao et al., 2012; Wang et al., 2019; Ma et al., 2017b; Chen et al., 2016). IMM-H007 decreased endothelial inflammation by suppressing NF-κB activity through the repression of IκBα degradation, NF-κB nuclear translocation, and JNK/AP1 signaling pathway (Yu et al., 2019). Notably, AMPK played a key role in regulating the expression of these genes, including ABCA1, suggesting that IMM-H007 promotes ABCA1 expression by suppressing ABCA1 degradation and enhancing AMPK expression. In addition, IMM-H007 suppressed cardiac fibrosis by enhancing AMPK and suppressing the TGFβ1/TGFβ type II receptor/Smad2/3 signaling pathway in mice (Wang et al., 2022b; Ge et al., 2019). IMM-H007 suppressed lipid accumulation, leukocyte trafficking, and macrophage infiltration in the liver by suppressing the AMPK/SREBP-1c, AMPK/acetyl-CoA carboxylase (ACC), and NF-κB/MCP-1 pathways in preclinical trials (Shi et al., 2017; Peng et al., 2019). IMM-H007 also improved the structure of the gut microbiota, including Firmicutes and Bacteroidetes, in hyperlipidemic hamsters (Li et al., 2018). IMM-H007 increased liver multiple gene expression in mice, including actinin alpha 2 (Actn2), Actn3, ATPase sarcoplasmic/endoplasmic reticulum Ca2+ transporting 1 (Atp2a1), calcium voltage-gated channel subunit alpha1 S (Cacna1s), calcium/calmodulin dependent protein kinase IV (Camk4), cAMP responsive element binding protein 5 (Creb5), cytochrome P450 family 17 subfamily A member 1 (Cyp17a1), growth arrest and DNA damage inducible alpha (Gadd45a), G protein subunit alpha L (Gnal), myosin heavy chain 7 (Myh7), myosin light chain 2 (Myl2), Myl3, Myl7, Myl11, myosin light chain kinase 2 (Mylk2), Mylk4, peroxisome proliferative activated receptor, gamma, coactivator 1 beta (Ppargc1b), protein phosphatase 1 regulatory subunit 3A (Ppp1r3a), glycogen phosphorylase, muscle associated (Pygm), ryanodine receptor 1 (Ryr1), solute carrier family 2 member 4 (Slc2a4), tribbles pseudokinase 3 (Trib3), and titin (Ttn) (Ma et al., 2017a). These results suggest that the off-target effects of IMM-H007 are relatively obvious, and whether IMM-H007 will cause toxic effects in clinical trials is unclear.

#### 4.1.3 RG7273

RG7273 (also named RG-7273) is a specific ABCA1 agonist that has entered phase 1 clinical trials. However, the development of RG7273 was discontinued on 12 April 2012 (Adisinsight, 2023; Zhao et al., 2013). The role of RG7273 in atherosclerosis models has not been investigated.

#### 4.1.4 R3R-01

R3R-01 (also named R3R01, RG-7273) is a specific ABCA1 agonist and entered into phase 2 clinical trials by River 3 Renal Corp. R3R-01 increases ABCA1 expression and may reduce kidney damage by reducing fat levels in the kidney (NephCure, 2024; Reiterova and Tesar, 2023). However, no further information about R3R01 has been reported, and its role in atherosclerosis models has also not been investigated.

### 4.2 FLNA

The diagnostic value of FLNA in emphysema (NCT05550844) and hepatocellular carcinoma (HCC, NCT03081637) is being investigated in clinical trials. Sumifilam (also named PTI-125 and simufilam), a small molecule antagonist, entered phase 3 for the treatment of mild-to-moderate AD (Wang et al., 2017; Wang et al., 2020; Zhang et al., 2020). However, the results of sumifilam in Phase 2 for the treatment of AD did not meet the primary endpoint on 15 May 2020 (Cassava Sciences, 2020). Notably, this result may have been masked by the high variability in the levels of disease biomarkers. Phase 3 trials of sumifilam for the treatment of AD are ongoing on 8 February 2023 (Cassava Sciences, 2023). The role of sumifilam in atherosclerosis models has not been investigated. More studies are needed to confirm the feasibility of FLNA as a target for drug development.

### 4.3 LRP6

#### 4.3.1 BI-905677

LRP6 is a promising target for disease diagnosis and drug development. Specifically, LRP6 combined with Klotho may be a prognostic biomarker of gastric adenocarcinoma and is being tested in clinical trials (NCT05293535). BI-905677, an LRP5 and LRP6 bipatopic nanobody inhibitor, completely blocked the binding of Wnt ligands to LRP5/LRP6. BI-905677 has been developed for the treatment of solid tumors in phase 1 clinical trials (Bayle et al., 2021). BI-905677 exhibited antitumor activity in preclinical trials, such as the ring finger protein 43 (RNF43) mutation tumor model and R-spondin 1 (RSPO) fusion tumor model. BI-905677 in combination with immune checkpoint inhibitors (such as anti-PD-1) also exhibited antitumor activity by inducing dendritic cell (DC) activation and T-cell infiltration in tumor tissues in preclinical trials (Vittoria Zinzalla et al., 2019). In phase 1 clinical trials on 8–13 April 2022, BI-905677 was well tolerated, and the maximum tolerated dose (MTD) was 2.8 mg/kg q3w. The incidence of grade 3 or higher adverse events (AEs), including vomiting, hyponatremia, anemia, diarrhea, abdominal pain, nausea, hypokalemia, pain, and increasing alkaline phosphatase (5%), was 51% (19/37). The best effect of BI-905677 is to stabilize the disease with a value of 35% (13/37) (Elena Élez et al., 2022). The phase 1 clinical trials of BI-905677 were terminated on 17 March 2023 (NCT03604445). Information on BI-905677 was also removed from Boehringer Ingelheim’s website (originator). These results suggest that BI-905677 is safety but moderately effective in cancer. However, the safety and effectiveness of BI-905677 in atherosclerosis have not been investigated.

#### 4.3.2 SZN-1326

SZN-1326, a bispecific tetravalent IgG1 molecule, is an Fzd5-and LRP6-specific Wnt mimetic that has been tested in clinical trials for the treatment of moderate to severe ulcerative colitis (UC) (Phase 1/1b, ACTRN12622000344796). SZN-1326 was derived from SZN-1326-p (Xie et al., 2022). In preclinical trials, SZN-132 inhibited colitis by promoting epithelial cell healing and reducing inflammatory cell infiltration (Xie et al., 2021a; CanaleComm, 2022). However, the phase 1 trial of SZN-1326 in inflammatory bowel disease (IBD) was suspended due to elevated liver enzymes (such as alanine transaminase and aspartate transaminase), which suggests that SZN-1326 may induce liver damage (Terry, 2022; Adisinsight, 2024). Notably, liver enzyme elevations were detected only in healthy volunteers and not in healthy participants. Total bilirubin, which is a signal of liver and bile duct damage, was not increased in participants. Moreover, no liver damage was detected, which suggests that increased liver enzymes may break down on their own (Terry, 2022). However, the role of SZN-1326 in atherosclerosis models has not been investigated. More studies are needed.

### 4.4 SOX2

STEMVAC is a multiantigen, multiepitope Th1 selective deoxyribonucleic acid (DNA) plasmid-based vaccine that targets SOX2, CD105, Y-box binding protein 1 (Yb-1), cadherin 3 (CDH3), and the MDM2 proto-oncogene (MDM2) and is being developed in clinical trials for the treatment of cancer (Phase 1/2), which suggests that SOX2 is a promising target for drug development (Higgins et al., 2016; Disis et al., 2022). In preclinical trials, STEMVAC was shown to be safe and to suppress tumor growth (Higgins et al., 2016). In combination with the adjuvant sargramostim, STEMVAC in patients with advanced HER2-negative breast cancer was found to be safe and to trigger a high level of sustained type I T-cell response in phase 1 clinical trials (Disis et al., 2022). The most common AEs were injection site reactions, influenza-like syndrome, transient leukopenia, and lymphocytopenia (Disis et al., 2022). Sargramostim is a yeast-derived recombinant human granulocyte-macrophage colony-stimulating factor (rhu GM-CSF). Sargramostim has been used for the treatment of acute radiation syndrome, bone marrow disorders, neutropenia, pneumococcal infections, and stem cell mobilization (Sargramostim, 2006; Lazarus et al., 2022; Tarhini et al., 2021). Clinical trials of sargramostim in other diseases, such as acute hypoxia, Alzheimer’s disease, chronic lymphocytic leukemia, hematological malignancies, malignant melanoma, mycobacterial infections, prostate cancer, and skin cancer, have also been conducted (AdisInsight, 2025). However, clinical trials of sargramostim in breast cancer have not been conducted. More studies are needed to confirm the effectiveness of STEMVAC in combination with sargramostim for preventing or treating breast cancer. In addition, the role of STEMVAC in atherosclerosis models has not been investigated.

Taken together, ARL4C-mediated genes, such as ABCA1, FLNA, LRP6, and SOX2, were the promising target genes for drug development (Table 2). Targeting these genes could improve the success rate of drug development into clinical trials and may be the first-in-class drug. However, to our knowledge, no specific agents have entered into clinical trials by targeting another ARL4C-mediated gene, including ALDH1A3, ARF6, ENHO, OSBPL5, and Snail2. More studies are needed to confirm the feasibility of these genes as a target for drug development. In addition, ARL4C is a promising biomarker for the diagnosis of renal cancer, gastric cancer, colorectal cancer, and lung adenocarcinoma in preclinical and clinical trials (Wei et al., 2009; Xie et al., 2021b; Matsumoto et al., 2017; Kimura et al., 2020). However, more studies are needed to confirm its sensitivity, specificity, early stage, late stage, and prognosis. Downregulation of ARL4C with siRNA and antisense oligonucleotides (ASOs), such as ASO-1316, is investigated in preclinical research for the treatment of lung adenocarcinoma (Kimura et al., 2020), liver cancer (Harada et al., 2019), and colorectal cancer (Fujii et al., 2015). However, to our knowledge, no specific ARL4C agonists and inhibitors have entered into clinical trials. More studies are needed to confirm the feasibility of ALR4C as a target for drug development.

**TABLE 2:** The agents in clinical trials by targeting ABCA1, FLNA, LRP6, and SOX2. Type and Group were obtained by Adisinsight, Bing, Chinadrugtrials, ClinicalTrials, Glgoo, Pharnexcloud, PubChem Compound, Pubmed, and Zhihuiya. AD, Alzheimer’s disease; IV, intravenous; NSCLC, non-small cell lung cancer; SC, subcutaneous; TNBS, triple negative breast cancer; UC, Ulcerative colitis.

Names	Structure/PubChem CID	Target	Administration	Status/Date	Diseases	Developer/Website	Patent	References
CS-6253	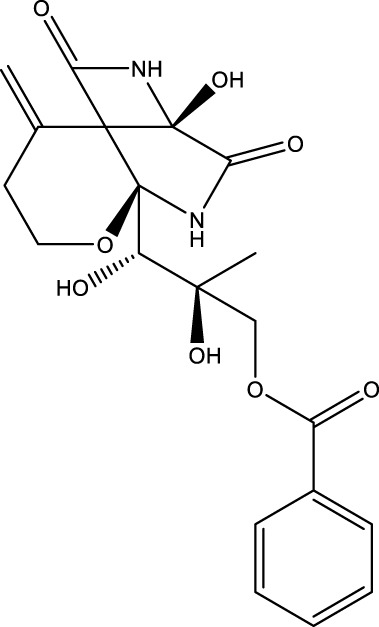 PubChem CID, 91618023; CAS, 37134-40-0 (FDA GSRS).	ABCA1 and apoE.	IV	Early Phase 1 (Recruiting on 31 January 2024)	AD	Artery Therapeutics, Inc. (https://www.arterytx.com/)	WO2011079214 (A1)	NCT05965414, (AdisInsight, 15 September 2022)
IMM-H007	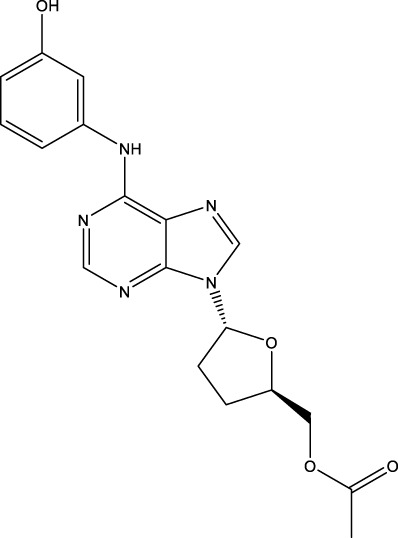 PubChem CID, 154730591	ABCA1, ABCG1, AMPK, eNOS, iNOS, LCAT, LOX-1, NF-κB, and TGFβ1.	Oral	Phase 1 (Recruiting on 16 March 2022)	Dyslipidemias	Tasly Pharmaceutical Group Co. Ltd. (https://en.taslypharma.com/)	WO2010040286 (A1)	CTR20220514 (Chinadrugtrials), (Huang et al., 2015; Gao et al., 2019; Zhao et al., 2012; Wang et al., 2019; Ma et al., 2017b; Chen et al., 2016; Yu et al., 2019)
RG7273	Structure not disclosed	ABCA1	Unknown	Phase 1 (Terminated on 12 April 2012)	Dyslipidemias	Roche Holding AG (https://www.roche.com/)	Unknown	Adisinsight (2023); Zhao et al. (2013)
R3R-01	Small molecule and structure not disclosed	ABCA1	Oral	Phase 2 (Recruiting on 15 June 2022)	Alport Syndrome, Focal Segmental Glomerulosclerosis	River 3 Renal Corp (Website: unknown)	WO2023039063 (A1)	NCT05267262
Sumifilam	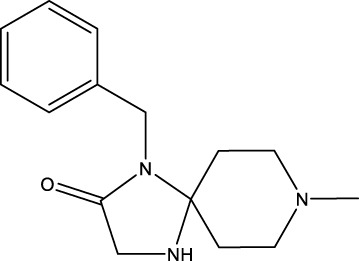 PubChem CID, 46195331; CAS, 1224591-33-6 (FDA GSRS).	FLNA	Oral	Phase 1 (Completed on 10 May 2021)	Healthy Volunteers	Cassava Sciences, Inc.	WO2014011917 (A2)	NCT03784300
[14C]-simufilam		FLNA	Oral	Phase 1 (Completed on 29 April 2024)	Healthy Volunteers			NCT06195319
Sumifilam		FLNA	Oral	Phase 1 (the Phase 3 oral tablet VS the Phase 2 oral tablet, completed on 22 August 2023)	Healthy Volunteers			NCT04932655
Sumifilam		FLNA	Unknown	Phase 1 (Not yet recruiting on 2 May 2024)	Moderate Hepatic Impairment			NCT05352763
Sumifilam		FLNA	Oral, twice a day, 28 days	Phase 2a (Completed on July 2021)	Mild-to-moderate AD			NCT03748706, 32920628
Sumifilam		FLNA	Oral, twice a day, 24 months	Phase 2b (Completed on September 2021)	Mild-to-moderate AD			NCT04079803, 33188449
Sumifilam		FLNA	Oral, twice a day	Phase 2 (Completed on 26 December 2023)	Mild-to-moderate AD			NCT04388254
Sumifilam		FLNA	Oral, twice a day	Phase 2 (Active, not recruiting on 8 January 2024)	Mild-to-moderate AD			NCT05352763
Sumifilam		FLNA	Oral, twice a day, 52 weeks	Phase 3 (Active, not recruiting on 26 January 2024)	Mild-to-moderate AD			NCT04994483
Sumifilam		FLNA	Oral, twice a day, 52 weeks	Phase 3 (Enrolling by invitation on 25 April 2024)	Mild-to-moderate AD			NCT05575076
Sumifilam		FLNA	Oral, twice a day, 76 weeks	Phase 3 (Active, not recruiting on 26 January 2024)	Mild-to-moderate AD			NCT05026177
BI-905677	Biparatopic nanobody	LRP5 and LRP6	IV	Phase 1 (Terminated on 4 March 2024)	Solid tumors	Boehringer Ingelheim International GmbH (https://www.boehringer-ingelheim.com/)	Unknown	NCT03604445
SZN-1326	Bispecific tetravalent IgG1 molecule	LRP6 and FZD5	SC, IV	Phase 1b (Suspended due to the liver enzyme elevations on 15 Nov ember 2022)	Moderate to severe UC	Surrozen Inc. (https://www.surrozen.com/)	WO2019124951 (A1)	Terry, 2022; Adisinsight (2024)
STEMVAC	Polyepitope Plasmid DNA Vaccine	CD105, CDH3, MDM2, SOX2, and Yb-1	Sargramostim intradermally	Phase 2 (Recruiting on 8 May 2024)	1 Early stage TNBS	National Cancer Institute (NCI) and University of Washington	Unknown	NCT05455658
STEMVAC			Sargramostim intradermally	Phase 2 (Recruiting on 2 April 2024)	2 Stage IV nonsquamous NSCLC			NCT05242965
STEMVAC			Sargramostim intradermally	Phase 1 (Active, not recruiting on 13 February 2024)	3 HER2-negative stage III-IV breast cancer			NCT02157051, (Disis et al., 2022; Higgins et al., 2016)

## 5 The regulation and mechanism of ARL4C by agents

**FIGURE 6:**
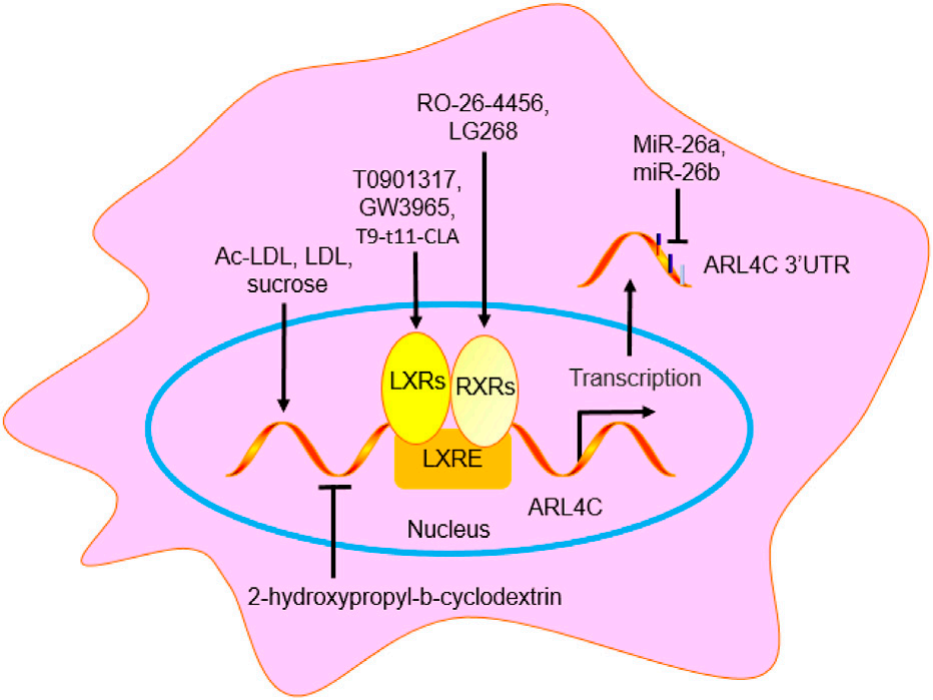
The regulation agent of ARL4C. GW3965, LG268, RO-26-4456, T0901317, and T9-t11-CLA promoted ARL4C expression by binding to LXRE. MiR-26a/b suppressed ARL4C expression by binding to ARL4C 3′UTR. Ac-LDL, LDL, and sucrose promoted ARL4C expression, while 2-hydroxypropyl-b-cyclodextrin suppressed ARL4C expression.

Many studies have shown that ARL4C is the only ARF (ARF1-6) and ARL family member (ARL1-6) whose mRNA is induced by LXR agonists (such as T0901317 and GW3965), retinoic X receptor (RXR) agonists (such as RO-26-4456 and LG268), and cholesterol-loading (Ac-LDL) in human monocyte-derived macrophages, RAW264.7 cells (a mouse macrophage line) and THP-1 cells (a human macrophage line) (Engel et al., 2004; Sun et al., 2012). LDL and LXR/RXR agonists also increased the ARL4C protein level by 1.8-fold and 3.2-fold, respectively, in HeLa cells, suggesting that the changes in the ARL4C protein level were consistent with those in the ARL4C mRNA level. However, 2-hydroxypropyl-β-cyclodextrin, which depletes cholesterol, reduces ARL4C expression (Engel et al., 2004; Helip-Wooley and Thoene, 2004). GW3965 increased ARL4C expression in the livers and spleens of C57BL/6 mice. In addition, sucrose and T9-t11-conjugated linoleic acid (CLA) increased ARL4C expression. T9-t11-CLA, which is the major isomer of CLA, a naturally occurring substance in dairy products and ruminant meat, reduced lipid accumulation by enhancing LXR expression *in vitro* and *in vivo*, suggesting that T9-t11-CLA is a novel potent LXR agonist (El Roz et al., 2013). Notably, the stimulatory effect of LXR/RXR agonists on ARL4C was greater than that on ABCA1 and ABCG1. Knockout of either LXRα or LXRβ significantly reduced ARL4C expression, while combined knockout resulted in the nonexpression of ARL4C, suggesting that both LXRα and LXRβ can independently regulate ARL4C expression (El Roz et al., 2013; Hong et al., 2011). MiR-26a (also named miR-26a-5p) and miR-26b (also named miR-26b-5p) suppressed cholesterol efflux to apoA-I by binding and suppressing ARL4C in RAW264.7 cells, THP-1 cells, and HepG2 cells (Sun et al., 2012). Taken together, many agents can regulate ARL4C expression, including LXR agonists (such as T0901317 and GW3965), RXR agonists (such as RO-26-4456 and LG268), Ac-LDL, LDL, 2-hydroxypropyl-b-cyclodextrin, sucrose, T9-t11-CLA, and miR-26 (Figure 6).

## 6 Patents related to ARL4C

The role of ARL4C in the diagnosis, prevention, treatment, and improvement of alzheimer’s disease and related neurodegenerative disorders was investigated by Evotec Neurosciences Gmbh (patent number: WO2004044592A1). Enhancing ARL4C reduced alzheimer’s disease development. The ARL4C variant can predict the effect of thiopurine therapy and is patented by Cedars-Sinai Medical Center (patent number: US20120190698A1). Inhibiting ARL4C (siRNA, antisense oligonucleotide, ribozyme, and siRNA expression vector) for the treatment of cancer (such as liver cancer, colon cancer, lung cancer, tongue cancer, and pancreatic cancer) was investigated by Osaka University NUC (patent number: JP06436477B2 and WO2020050307A1). ARL4C can be used as a prognostic biomarker for the survival of patients with pancreatic cancer treated with gemcitabine and has been patented by Acobiom (patent number: WO2016027029A2). The role of ARL4C in COVID-19 infection has been patented by Genuity Science, Inc. (patent number: WO2022240743A1 and WO2022240746A1). There are many other patents related to ARL4C (Table 3), such as short (or small) activating RNA (saRNA, patent number: JP2021035360A and JP2018512876A6), RNA encoding a therapeutic protein (patent number: US20190241633A1), immunotherapy (patent number: US20200157633A1, US20200016202A1, WO2017069958A2, and US20140073526A1), sudden cardiac event (patent number: AU2011227108A1), cardiac developmental (patent number: WO2018007525A2), dysregulated lipid metabolism (patent number: US20230132366A9 and US20200360375A1), pulmonary arterial hypertension (PAH, patent number: WO2017089593A1).

**TABLE 3:** Patents related to ARL4C.

Diseases	Function	Patent number
Alzheimer’s disease and neurodegenerative disorders	Diagnosis, prevention, treatment, and improvement	WO2004044592A1
Thiopurine therapy	Predict the effect	US20120190698A1
Cancer (including liver cancer, colon cancer, lung cancer, tongue cancer, and pancreatic cancer)	SiRNA, antisense oligonucleotide, and ribozyme	JP06436477B2 and WO2020050307A1
Pancreatic cancer	Prognostic biomarker	WO2016027029A2
Short activating RNA		JP2021035360A and JP2018512876A6
RNA encoding a therapeutic protein		US20190241633A1
Immunotherapy		US20200157633A1, US20200016202A1, WO2017069958A2, and US20140073526A1
Sudden cardiac event		AU2011227108A1
Cardiac developmental		WO2018007525A2
Dysregulated lipid metabolism		US20230132366A9 and US20200360375A1
Pulmonary arterial hypertension		WO2017089593A1

## 7 Conclusion

ARL4C promoted cholesterol efflux. ARL4C promoted ABCA1, ALDH1A3, ARF6, ENHO, FLNA, LRP6, OSBPL5, and Snail2 expression and reduced SOX2 expression. However, most of the regulatory mechanisms of ARL4C, except for ARF6, FLNA, and OSBPL5, are unclear. The main target gene of ARL4C is also unclear. ARL4C may exhibit antiatherosclerotic effects by enhancing ABCA1, ENHO, LRP6, and OSBPL5 expression but may exhibit proatherosclerotic effects by enhancing ARF6 and Snail2 expression. The pro-atherosclerosis or anti-atherosclerosis effects of many regulatory genes, such as FLNA and SOX2, depend on their location. Cell- or tissue-specific ARL4C localization may be an important inducer of its dual role in atherosclerosis. However, larger studies, such as those involving overexpression, deficiency, inhibition, knockout, GWAS, and exome sequencing in animal models, are needed to confirm whether it is proatherogenic or antiatherogenic. Many ARL4C downstream genes, including ABCA, FLNA, LRP6, and SOX2, are promising targets for drug development because many drugs have entered clinical trials. However, no specific agents targeting ABCA1 and LRP6 have entered phase 2 clinical trials. More studies are needed to confirm the development value of targeting ABCA1 and LRP6. The detection value of ARL4C downstream genes, such as ENHO FLNA and LRP6, is being investigated in clinical trials. ARL4C is also a promising biomarker for the diagnosis of cancer. However, more studies are needed to confirm its sensitivity, specificity, early stage, late stage, and prognosis. The downregulation of ARL4C with siRNAs and ASOs is being investigated in preclinical research. Therefore, ARL4C may be a potential target for disease diagnosis and therapeutic drug development. However, no ARL4C agonists or inhibitors have entered clinical trials. It is also necessary to consider whether regulating ARL4C can regulate downstream genes and which genes it regulates. The target specificity, target tissue expressivity, dosage, and toxicity should be considered in drug development. More studies are needed to confirm the development value of ARL4C as a target for disease diagnosis and drug development. With the progress of science and technology, the deepening of research, and the cooperation of scientific research, we believe that more scientists will study ARL4C and its downstream genes to identify potential biomarkers and novel therapeutic targets and drugs.

## Glossary

VSMCs: vascular smooth muscle cells
ABCA1: ATP binding cassette transporter A1
SR-B1: Scavenger receptor type B1
ApoA-I: Apolipoprotein A-I
HDL: High-density lipoprotein
ARL7: ARF-like 7
ARL4C: ADP-ribosylation factor-like 4C
TLAK: Lymphokine-activated T-killer
ENHO: Energy homeostasis associated
FLNA: Filamin-A
LRP6: Low-density lipoprotein receptor-related protein-6
OSBPL5: Oxysterol binding protein like 5
Snail2: Snail family zinc finger 2
SOX2: Sex-determining region Y-box 2
BMDMs: Bone marrow-derived macrophages
RCT: Reverse cholesterol transport
ER: Endoplasmic reticulum
TLR-4: Toll-like receptor-4
NF-kB: Nuclear factor kB
ANXA1: Annexin A1
PTB: Phosphotyrosine-binding domain
GULP1: Engulfment adaptor PTB domain containing 1
MEGF10: Multiple EGF-like domains 10
PtdSer: Phosphatidylserine
TG2: Transglutaminase 2
MMP2: Matrix metalloproteinase-2
LXRs: Liver X-receptors
VEGFR2: Vascular endothelial growth factor receptor-2
eNOS: Endothelial nitric oxide synthase
PDH: Pyruvate dehydrogenase
SIRT1: Silent information regulator sirtuin 1
PDK4: Pyruvate dehydrogenase kinase 4
PGC-1α: Peroxisome proliferator-activated receptor-γ coactivator-1α
CPT1B: Carnitine palmitoyltransferase 1B
FGD6: Faciogenital dysplasia 6
Cdc42: Cell division cycle 42
LTA: Lymphotoxin-α
agLDL: aggregated LDL
SREBP1/2: Sterol response element binding protein 1/2
DRP1: Dynamin-related protein 1
TFEB: Transcription factor EB
LEs/LYs: Late endosomes/lysosomes
H3K4Me3: Histone 3 lysine 4 trimethylation
HMGCR: HMG-CoA reductase
EMT: Epithelial-to-mesenchymal transition
EndMT: Endothelial-to-mesenchymal transition
COX-2: Cyclooxygenase-2
PGE2: Prostaglandin E2
iPSCs: Induced pluripotent stem cells
ASOs: Antisense oligonucleotides
AMPK: AMP-activated protein kinase
TGFβ1: Transforming growth factor β1
TNFα: Tumor necrosis factor-α
IL-1: Interleukin-1
MCP-1: Monocyte chemoattractant protein 1
NO: Nitric oxide
iNOS: Inducible NO synthase
LOX-1: Lectin-like oxidized LDL receptor-1
MPO: Myeloperoxidase
Arg-1: Arginase 1
LCAT: Lecithin-cholesterol acyltransferase
pAMPK: Phosphorylation of AMPK
PON1: Paraoxonase 1
ACC: Acetyl-CoA carboxylase
Actn2: Actinin alpha 2
Atp2a1: ATPase sarcoplasmic/endoplasmic reticulum Ca2+ transporting 1
Cacna1s: Calcium voltage-gated channel subunit alpha1 S
Camk4: Calcium/calmodulin dependent protein kinase IV
Creb5: cAMP responsive element binding protein 5
Cyp17a1: Cytochrome P450 family 17 subfamily A member 1
Gadd45a: Growth arrest and DNA damage inducible alpha
Gnal: G protein subunit alpha L
Myh7: Myosin heavy chain 7
Myl2: Myosin light chain 2
Mylk2: Myosin light chain kinase 2
Ppargc1b: Peroxisome proliferative activated receptor, gamma, coactivator 1 beta
Ppp1r3a: Protein phosphatase 1 regulatory subunit 3A
Pygm: Glycogen phosphorylase, muscle associated
Ryr1: Ryanodine receptor 1
Slc2a4: Solute carrier family 2 member 4
Trib3: Tribbles pseudokinase 3
Ttn: Titin
AD: Alzheimer’s disease
T2DM: Type 2 diabetes
HCC: Hepatocellular carcinoma
UC: Ulcerative colitis
IBD: Inflammatory bowel disease
RNF43: Ring finger protein 43
RSPO: R-spondin 1
DC: Dendritic cell
MTD: Maximum tolerated dose
AEs: Adverse events
DNA: Deoxyribonucleic acid
Yb-1: Y-box binding protein 1
CDH3: Cadherin 3
MDM2: MDM2 proto-oncogene
rhu GM-CSF: Recombinant human granulocyte-macrophage colony-stimulating factor

## References

[B1] ADISINSIGHT (2024). CS 6253. Available at: https://adis.springer.com/drugs/800050173 (Accessed September 27, 2024).

[B2] ADISINSIGHT (2023). RG 7273. Available at: https://adis.springer.com/drugs/800031594 (Accessed November 05, 2023).

[B4] ADISINSIGHT (2025). Sargramostim - bayer HealthCare pharmaceuticals/partner therapeutics. Available at: https://adisinsight.springer.com/drugs/800004168?bpIds=3000093925%2C3001592458&checksum=42c3a41b65e5fcc5722a660d8ec40958ae02aae7-1586786578832-a45dccf765662b475d25ed01b15b6367979854bdf911905893237a83e0d9ad4a05397e65a874c51567277828def432d0 (Accessed January 22, 2025).

[B5] ADISINSIGHT (2024). SZN 1326. Available at: https://adis.springer.com/drugs/800059234 (Accessed January 22, 2024).

[B6] Alrefaei A. F. Abu-Elmagd M. (2022). LRP6 receptor plays essential functions in development and human diseases. Genes (Basel) 13, 120. 10.3390/genes13010120 35052459 PMC8775365

[B7] Bandaru S. Ala C. Ekstrand M. Akula M. K. Pedrelli M. Liu X. (2020). Lack of RAC1 in macrophages protects against atherosclerosis. PLoS One 15, e0239284. 10.1371/journal.pone.0239284 32941503 PMC7498073

[B8] Bandaru S. Gronros J. Redfors B. Cil C. Pazooki D. Salimi R. (2015). Deficiency of filamin A in endothelial cells impairs left ventricular remodelling after myocardial infarction. Cardiovasc Res. 105, 151–159. 10.1093/cvr/cvu226 25344364

[B9] Bayle E. D. Svensson F. Atkinson B. N. Steadman D. Willis N. J. Woodward H. L. (2021). Carboxylesterase notum is a druggable target to modulate Wnt signaling. J. Med. Chem. 64, 4289–4311. 10.1021/acs.jmedchem.0c01974 33783220 PMC8172013

[B10] Bi X. Vitali C. Cuchel M. (2015). ABCA1 and inflammation: from animal models to humans. Arterioscler. Thromb. Vasc. Biol. 35, 1551–1553. 10.1161/ATVBAHA.115.305547 26109737

[B11] Bostrom K. I. Yao J. Guihard P. J. Blazquez-Medela A. M. Yao Y. (2016). Endothelial-mesenchymal transition in atherosclerotic lesion calcification. Atherosclerosis 253, 124–127. 10.1016/j.atherosclerosis.2016.08.046 27615595 PMC5064862

[B12] CANALECOMM (2022). Surrozen presents Data on lead therapeutic candidates at digestive disease week (DDW). Available at: https://www.biospace.com/article/releases/surrozen-presents-data-on-lead-therapeutic-candidates-at-digestive-disease-week-ddw-/?keywords=Digestive+Disease+Week (Accessed May 24, 2022).

[B13] CASSAVA SCIENCES, I (2020). Top-line results from a phase 2b study of PTI-125 in alzheimer’s disease does not meet primary endpoint. Available at: https://www.globenewswire.com/news-release/2020/05/15/2034228/8339/en/Top-line-Results-from-a-Phase-2b-Study-of-PTI-125-in-Alzheimer-s-Disease-Does-Not-Meet-Primary-Endpoint.html (Accessed May 15, 2020).

[B14] CASSAVA SCIENCES, I. (2023). Cassava sciences announces patient enrollment update for phase 3 studies of simufilam for the treatment of alzheimer’s disease. Available at: https://www.globenewswire.com/news-release/2023/02/08/2604083/0/en/Cassava-Sciences-Announces-Patient-Enrollment-Update-for-Phase-3-Studies-of-Simufilam-for-the-Treatment-of-Alzheimer-s-Disease.html (Accessed February 08, 2023).

[B15] Chen B. Li J. Zhu H. (2016). AMP-activated protein kinase attenuates oxLDL uptake in macrophages through PP2A/NF-κB/LOX-1 pathway. Vasc. Pharmacol. 85, 1–10. 10.1016/j.vph.2015.08.012 26297684

[B16] Chen Q. Fu W. J. Tang X. P. Wang L. Niu Q. Wang S. (2021a). ADP-Ribosylation Factor like GTPase 4C (ARL4C) augments stem-like traits of glioblastoma cells by upregulating ALDH1A3. J. Cancer 12, 818–826. 10.7150/jca.45052 33403039 PMC7778538

[B17] Chen W. Li L. Wang J. Zhang R. Zhang T. Wu Y. (2021b). The ABCA1-efferocytosis axis: a new strategy to protect against atherosclerosis. Clin. Chim. Acta 518, 1–8. 10.1016/j.cca.2021.02.025 33741356

[B18] Chen W. Wang S. Xing D. (2021c). New horizons for the roles and association of APE1/ref-1 and ABCA1 in atherosclerosis. J. Inflamm. Res. 14, 5251–5271. 10.2147/JIR.S330147 34703267 PMC8526300

[B19] Chen W. Wu Y. Lu Q. Wang S. Xing D. (2020a). Endogenous ApoA-I expression in macrophages: a potential target for protection against atherosclerosis. Clin. Chim. Acta 505, 55–59. 10.1016/j.cca.2020.02.025 32092318

[B20] Chen W. Xing J. Liu X. Wang S. Xing D. (2021d). The role and transformative potential of IL-19 in atherosclerosis. Cytokine Growth Factor Rev. 62, 70–82. 10.1016/j.cytogfr.2021.09.001 34600839

[B21] Chen W. Zhang S. Wu J. Ye T. Wang S. Wang P. (2020b). Butyrate-producing bacteria and the gut-heart axis in atherosclerosis. Clin. Chim. Acta 507, 236–241. 10.1016/j.cca.2020.04.037 32376324

[B22] Chen W. Zhong Y. Feng N. Guo Z. Wang S. Xing D. (2021e). New horizons in the roles and associations of COX-2 and novel natural inhibitors in cardiovascular diseases. Mol. Med. 27, 123. 10.1186/s10020-021-00358-4 34592918 PMC8482621

[B23] Chiang T. S. Wu H. F. Lee F. S. (2017). ADP-ribosylation factor-like 4C binding to filamin-A modulates filopodium formation and cell migration. Mol. Biol. Cell 28, 3013–3028. 10.1091/mbc.E17-01-0059 28855378 PMC5662259

[B24] Desita S. R. Hariftyani A. S. Jannah A. R. Setyobudi A. K. Oktaviono Y. H. (2022). PCSK9 and LRP6: potential combination targets to prevent and reduce atherosclerosis. J. Basic Clin. Physiol. Pharmacol. 33, 529–534. 10.1515/jbcpp-2021-0291 35429418

[B25] Disis M. Liu Y. Stanton S. Gwin W. Coveler A. Liao J. (2022). 546 A phase I dose escalation study of STEMVAC, a multi-antigen, multi-epitope Th1 selective plasmid-based vaccine, targeting stem cell associated proteins in patients with advanced breast cancer. J. Immunother. Cancer 10, A571. 10.1136/jitc-2022-sitc2022.0546

[B26] Dissmore T. Seye C. I. Medeiros D. M. Weisman G. A. Bradford B. Mamedova L. (2016). The P2Y2 receptor mediates uptake of matrix-retained and aggregated low density lipoprotein in primary vascular smooth muscle cells. Atherosclerosis 252, 128–135. 10.1016/j.atherosclerosis.2016.07.927 27522265 PMC5060008

[B27] Elena Élez H.-J. L. Maja D. E. JONGE Yaeger RONA Toshihiko D. O. I. Pronk LINDA Teufel MICHAEL (2022). A phase I, open-label, dose-escalation study investigating a low-density lipoprotein receptor-related protein (LRP) 5/6 inhibitor, BI 905677, in patients with advanced solid tumors. Cancer Res. 82, CT514.

[B28] El Roz A. Bard J. M. Huvelin J. M. Nazih H. (2013). The anti-proliferative and pro-apoptotic effects of the trans9, trans11 conjugated linoleic acid isomer on MCF-7 breast cancer cells are associated with LXR activation. Prostagl. Leukot. Essent. Fat. Acids 88, 265–272. 10.1016/j.plefa.2012.12.006 23375583

[B29] Engel T. Lueken A. Bode G. Hobohm U. Lorkowski S. Schlueter B. (2004). ADP-ribosylation factor (ARF)-like 7 (ARL7) is induced by cholesterol loading and participates in apolipoprotein AI-dependent cholesterol export. FEBS Lett. 566, 241–246. 10.1016/j.febslet.2004.04.048 15147902

[B30] Escate R. Padro T. Borrell-Pages M. Suades R. Aledo R. Mata P. (2017). Macrophages of genetically characterized familial hypercholesterolaemia patients show up-regulation of LDL-receptor-related proteins. J. Cell Mol. Med. 21, 487–499. 10.1111/jcmm.12993 27680891 PMC5323824

[B31] Fiola-Masson E. Artigalas J. Campbell S. Claing A. (2022). Activation of the GTPase ARF6 regulates invasion of human vascular smooth muscle cells by stimulating MMP14 activity. Sci. Rep. 12, 9532. 10.1038/s41598-022-13574-7 35680971 PMC9184495

[B32] Fujii S. Ishibashi T. Kokura M. Fujimoto T. Matsumoto S. Shidara S. (2022). RAF1-MEK/ERK pathway-dependent ARL4C expression promotes ameloblastoma cell proliferation and osteoclast formation. J. Pathol. 256, 119–133. 10.1002/path.5814 34622442

[B33] Fujii S. Matsumoto S. Nojima S. Morii E. Kikuchi A. (2015). Arl4c expression in colorectal and lung cancers promotes tumorigenesis and may represent a novel therapeutic target. Oncogene 34, 4834–4844. 10.1038/onc.2014.402 25486429

[B34] Gao F. Qian Y. J. Chen F. H. Zhu H. B. (2019). Comparative analysis of stimulation and binding characteristics of adenosine analogs to AMP-activated protein kinase. J. Asian Nat. Prod. Res. 21, 916–927. 10.1080/10286020.2018.1484454 30187782

[B35] Ge W. Zhang W. Gao R. Li B. Zhu H. Wang J. (2019). IMM-H007 improves heart function via reducing cardiac fibrosis. Eur. J. Pharmacol. 857, 172442. 10.1016/j.ejphar.2019.172442 31181209

[B36] Griffiths G. S. Grundl M. Allen J. S. Matter M. L. (2011). R-Ras interacts with filamin a to maintain endothelial barrier function. J. Cell Physiol. 226, 2287–2296. 10.1002/jcp.22565 21660952 PMC3080452

[B37] Grimbert P. Valanciute A. Audard V. Lang P. Guellaen G. Sahali D. (2004). The Filamin-A is a partner of Tc-mip, a new adapter protein involved in c-maf-dependent Th2 signaling pathway. Mol. Immunol. 40, 1257–1261. 10.1016/j.molimm.2003.11.035 15128042

[B38] Guo J. Hao C. Wang C. Li L. (2018). Long noncoding RNA PVT1 modulates hepatocellular carcinoma cell proliferation and apoptosis by recruiting EZH2. Cancer Cell Int. 18, 98. 10.1186/s12935-018-0582-3 30008615 PMC6042336

[B39] Hafiane A. Johansson J. O. Genest J. (2019). ABCA1 agonist mimetic peptide CS-6253 induces microparticles release from different cell types by ABCA1-efflux-dependent mechanism. Can. J. Cardiol. 35, 770–781. 10.1016/j.cjca.2019.02.018 31151713

[B40] Han J. S. Hino K. Li W. Reyes R. V. Canales C. P. Miltner A. M. (2020). CRL5-dependent regulation of the small GTPases ARL4C and ARF6 controls hippocampal morphogenesis. Proc. Natl. Acad. Sci. U. S. A. 117, 23073–23084. 10.1073/pnas.2002749117 32873638 PMC7502717

[B41] Han S. Xu S. Zhou J. Qiao A. Boriboun C. Ma W. (2019). Sam68 impedes the recovery of arterial injury by augmenting inflammatory response. J. Mol. Cell Cardiol. 137, 82–92. 10.1016/j.yjmcc.2019.10.003 31639388 PMC6889069

[B42] Harada T. Matsumoto S. Hirota S. Kimura H. Fujii S. Kasahara Y. (2019). Chemically modified antisense oligonucleotide against ARL4C inhibits primary and metastatic liver tumor growth. Mol. Cancer Ther. 18, 602–612. 10.1158/1535-7163.MCT-18-0824 30647122

[B43] Helip-Wooley A. Thoene J. G. (2004). Sucrose-induced vacuolation results in increased expression of cholesterol biosynthesis and lysosomal genes. Exp. Cell Res. 292, 89–100. 10.1016/j.yexcr.2003.09.003 14720509

[B44] Higgins D. M. Childs J. S. Salazar L. Disis M. (2016). Abstract OT1-01-01: A phase I trial of the safety and immunogenicity of a multiple antigen vaccine (STEMVAC) in HER2 negative advanced stage breast cancer patients. Cancer Res. 76 10.1158/1538-7445.sabcs15-ot1-01-01

[B45] Hofmann I. Thompson A. Sanderson C. M. Munro S. (2007). The Arl4 family of small G proteins can recruit the cytohesin Arf6 exchange factors to the plasma membrane. Curr. Biol. 17, 711–716. 10.1016/j.cub.2007.03.007 17398095

[B46] Hong C. Walczak R. Dhamko H. Bradley M. N. Marathe C. Boyadjian R. (2011). Constitutive activation of LXR in macrophages regulates metabolic and inflammatory gene expression: identification of ARL7 as a direct target. J. Lipid Res. 52, 531–539. 10.1194/jlr.M010686 21187453 PMC3035689

[B47] Hua T. N. M. Namkung J. Phan A. N. H. Vo V. T. A. Kim M. K. Jeong Y. (2018). PPARgamma-mediated ALDH1A3 suppression exerts anti-proliferative effects in lung cancer by inducing lipid peroxidation. J. Recept Signal Transduct. Res. 38, 191–197. 10.1080/10799893.2018.1468781 29873276

[B48] Huang L. Fan B. Ma A. Shaul P. W. Zhu H. (2015). Inhibition of ABCA1 protein degradation promotes HDL cholesterol efflux capacity and RCT and reduces atherosclerosis in mice. J. Lipid Res. 56, 986–997. 10.1194/jlr.M054742 25761370 PMC4409288

[B49] Hu Q. Masuda T. Sato K. Tobo T. Nambara S. Kidogami S. (2018). Identification of ARL4C as a peritoneal dissemination-associated gene and its clinical significance in gastric cancer. Ann. Surg. Oncol. 25, 745–753. 10.1245/s10434-017-6292-6 29270876

[B50] Ishikawa S. Nagai Y. Masuda T. Koga Y. Nakamura T. Imamura Y. (2010). The role of oxysterol binding protein-related protein 5 in pancreatic cancer. Cancer Sci. 101, 898–905. 10.1111/j.1349-7006.2009.01475.x 20128820 PMC11159417

[B51] Jacobs S. Schilf C. Fliegert F. Koling S. Weber Y. Schurmann A. (1999). ADP-ribosylation factor (ARF)-like 4, 6, and 7 represent a subgroup of the ARF family characterization by rapid nucleotide exchange and a nuclear localization signal. FEBS Lett. 456, 384–388. 10.1016/s0014-5793(99)00759-0 10462049

[B52] Jaiswal S. Rajnikanth P. S. Thakur S. Deepak P. Anand S. (2021). A review on novel ligand targeted delivery for cardiovascular disorder. Curr. Drug Deliv. 18, 1094–1104. 10.2174/1567201818666210301095046 33645481

[B53] Kang S. (2020). Low-density lipoprotein receptor-related protein 6-mediated signaling pathways and associated cardiovascular diseases: diagnostic and therapeutic opportunities. Hum. Genet. 139, 447–459. 10.1007/s00439-020-02124-8 32076828

[B54] Karimian M. Nouri N. Ghasemi L. V. Mohammadi A. H. Behjati M. (2023). Administration of stem cells against cardiovascular diseases with a focus on molecular mechanisms: current knowledge and prospects. Tissue Cell 81, 102030. 10.1016/j.tice.2023.102030 36709696

[B55] Kimura K. Matsumoto S. Harada T. Morii E. Nagatomo I. Shintani Y. (2020). ARL4C is associated with initiation and progression of lung adenocarcinoma and represents a therapeutic target. Cancer Sci. 111, 951–961. 10.1111/cas.14303 31925985 PMC7060486

[B56] Kram H. Prokop G. Haller B. Gempt J. Wu Y. Schmidt-Graf F. (2022). Glioblastoma relapses show increased markers of vulnerability to ferroptosis. Front. Oncol. 12, 841418. 10.3389/fonc.2022.841418 35530303 PMC9071304

[B57] Kumar D. Narang R. Saluja D. Srivastava K. (2022). Functional association of miR-133b and miR-21 through novel gene targets ATG5, LRP6 and SGPP1 in coronary artery disease. Mol. Diagn Ther. 26, 655–664. 10.1007/s40291-022-00615-0 36197604

[B58] Lazarus H. M. Mcmanus J. Gale R. P. (2022). Sargramostim in acute radiation syndrome. Expert Opin. Biol. Ther. 22, 1345–1352. 10.1080/14712598.2022.2143261 36325797

[B59] Ledard N. Liboz A. Blondeau B. Babiak M. Moulin C. Vallin B. (2020). Slug, a cancer-related transcription factor, is involved in vascular smooth muscle cell transdifferentiation induced by platelet-derived growth factor-BB during atherosclerosis. J. Am. Heart Assoc. 9, e014276. 10.1161/JAHA.119.014276 31959031 PMC7033846

[B60] Li D. Shao N. Y. Moonen J. R. Zhao Z. Shi M. Otsuki S. (2021). ALDH1A3 coordinates metabolism with gene regulation in pulmonary arterial hypertension. Circulation 143, 2074–2090. 10.1161/CIRCULATIONAHA.120.048845 33764154 PMC8289565

[B61] Li L. Xie W. Zheng X. L. Yin W. D. Tang C. K. (2016). A novel peptide adropin in cardiovascular diseases. Clin. Chim. Acta 453, 107–113. 10.1016/j.cca.2015.12.010 26683354

[B62] Li T. Sun S. Zhang J. Qu K. Yang L. Ma C. (2018). Beneficial metabolic effects of 2',3',5'-triacetyl-N(6)-(3-hydroxylaniline) adenosine in multiple biological matrices and intestinal flora of hyperlipidemic hamsters. J. Proteome Res. 17, 2870–2879. 10.1021/acs.jproteome.8b00330 29925242

[B63] Ma A. Wang D. An Y. Fang W. Zhu H. (2017a). Comparative transcriptomic analysis of mice liver treated with different AMPK activators in a mice model of atherosclerosis. Oncotarget 8, 16594–16604. 10.18632/oncotarget.15027 28178661 PMC5369987

[B64] Ma A. Wang J. Yang L. An Y. Zhu H. (2017b). AMPK activation enhances the anti-atherogenic effects of high density lipoproteins in apoE(-/-) mice. J. Lipid Res. 58, 1536–1547. 10.1194/jlr.M073270 28611100 PMC5538277

[B65] Mahmud S. Alam S. Emon N. U. Boby U. H. Ahmed F. Monjur-Al-Hossain A. S. M. (2022). Opportunities and challenges in stem cell therapy in cardiovascular diseases: position standing in 2022. Saudi Pharm. J. 30, 1360–1371. 10.1016/j.jsps.2022.06.017 36249945 PMC9563042

[B66] Mansfield C. Zhao M. T. Basu M. (2022). Translational potential of hiPSCs in predictive modeling of heart development and disease. Birth Defects Res. 114, 926–947. 10.1002/bdr2.1999 35261209 PMC9458775

[B67] Matsumoto S. Fujii S. Kikuchi A. (2017). Arl4c is a key regulator of tubulogenesis and tumourigenesis as a target gene of Wnt-β-catenin and growth factor-Ras signalling. J. Biochem. 161, 27–35. 10.1093/jb/mvw069 28053143

[B68] Matsuo M. (2022). ABCA1 and ABCG1 as potential therapeutic targets for the prevention of atherosclerosis. J. Pharmacol. Sci. 148, 197–203. 10.1016/j.jphs.2021.11.005 35063134

[B69] Mocnik M. Marcun Varda N. (2022). Current knowledge of selected cardiovascular biomarkers in pediatrics: kidney injury molecule-1, salusin-α and -β, uromodulin, and adropin. Child. (Basel) 9, 102. 10.3390/children9010102 PMC8774650 35053727

[B70] Muhammed A. A. Eid R. Mohammed W. S. Abdel-Fadeil M. R. (2022). An association between adropin hormone and total testosterone in obese men: a case-control study. BMC Endocr. Disord. 22, 192. 10.1186/s12902-022-01102-7 35897011 PMC9327160

[B71] Mukhamedova N. Hoang A. Cui H. L. Carmichael I. Fu Y. Bukrinsky M. (2016). Small GTPase ARF6 regulates endocytic pathway leading to degradation of ATP-binding cassette transporter A1. Arterioscler. Thromb. Vasc. Biol. 36, 2292–2303. 10.1161/ATVBAHA.116.308418 27758770 PMC5123748

[B72] NEPHCURE (2024). R3R01: AS-FSGS. Available at: https://nephcure.org/trials/r3r-asfsgs-trial-amsterdam-netherlands/.

[B73] Niepolski L. Grzegorzewska A. E. (2016). Salusins and adropin: new peptides potentially involved in lipid metabolism and atherosclerosis. Adv. Med. Sci. 61, 282–287. 10.1016/j.advms.2016.03.007 27128818

[B74] Noveir S. D. Kerman B. E. Xian H. Meuret C. Smadi S. Martinez A. E. (2022). Effect of the ABCA1 agonist CS-6253 on amyloid-β and lipoprotein metabolism in cynomolgus monkeys. Alzheimers Res. Ther. 14, 87. 10.1186/s13195-022-01028-1 35751102 PMC9229758

[B75] Peng X. Li J. Wang M. Qu K. Zhu H. (2019). A novel AMPK activator improves hepatic lipid metabolism and leukocyte trafficking in experimental hepatic steatosis. J. Pharmacol. Sci. 140, 153–161. 10.1016/j.jphs.2019.05.008 31253430

[B76] Rajamannan N. M. (2011). The role of Lrp5/6 in cardiac valve disease: LDL-density-pressure theory. J. Cell Biochem. 112, 2222–2229. 10.1002/jcb.23182 21590710 PMC3927739

[B77] Reiterova J. Tesar V. (2023). Current and future therapeutical options in alport syndrome. Int. J. Mol. Sci. 24, 5522. 10.3390/ijms24065522 36982595 PMC10056269

[B78] Santos N. C. Girik V. Nunes-Hasler P. (2020). ORP5 and ORP8: sterol sensors and phospholipid transfer proteins at membrane contact sites? Biomolecules 10, 928. 10.3390/biom10060928 32570981 PMC7356933

[B79] Sargramostim (2006). Drugs and lactation database (LactMed(R)). Bethesda, MD: National Institute of Child Health and Human Development.

[B80] Sedgwick A. Olivia Balmert M. D'Souza-Schorey C. (2018). The formation of giant plasma membrane vesicles enable new insights into the regulation of cholesterol efflux. Exp. Cell Res. 365, 194–207. 10.1016/j.yexcr.2018.03.001 29522754 PMC6652231

[B81] Sharma A. Batra J. Stuchlik O. Reed M. S. Pohl J. Chow V. T. K. (2020). Influenza A virus nucleoprotein activates the JNK stress-signaling pathway for viral replication by sequestering host filamin A protein. Front. Microbiol. 11, 581867. 10.3389/fmicb.2020.581867 33101257 PMC7546217

[B82] Shi H. Liang M. Chen W. Sun X. Wang X. Li C. (2018). Human induced pluripotent stem cell-derived mesenchymal stem cells alleviate atherosclerosis by modulating inflammatory responses. Mol. Med. Rep. 17, 1461–1468. 10.3892/mmr.2017.8075 29257199 PMC5780084

[B83] Shi H. Wang Q. Yang L. Xie S. Zhu H. (2017). IMM-H007, a new therapeutic candidate for nonalcoholic fatty liver disease, improves hepatic steatosis in hamsters fed a high-fat diet. FEBS Open Bio 7, 1379–1391. 10.1002/2211-5463.12272 PMC5586352 28904866

[B84] Sun D. Zhang J. Xie J. Wei W. Chen M. Zhao X. (2012). MiR-26 controls LXR-dependent cholesterol efflux by targeting ABCA1 and ARL7. FEBS Lett. 586, 1472–1479. 10.1016/j.febslet.2012.03.068 22673513

[B85] Szanto M. Gupte R. Kraus W. L. Pacher P. Bai P. (2021). PARPs in lipid metabolism and related diseases. Prog. Lipid Res. 84, 101117. 10.1016/j.plipres.2021.101117 34450194

[B86] Tarhini A. A. Joshi I. Garner F. (2021). Sargramostim and immune checkpoint inhibitors: combinatorial therapeutic studies in metastatic melanoma. Immunotherapy 13, 1011–1029. 10.2217/imt-2021-0119 34157863

[B87] Tavano R. Contento R. L. Baranda S. J. Soligo M. Tuosto L. Manes S. (2006). CD28 interaction with filamin-A controls lipid raft accumulation at the T-cell immunological synapse. Nat. Cell Biol. 8, 1270–1276. 10.1038/ncb1492 17060905

[B88] Terry M. (2022). Surrozen pauses phase I trial following liver enzyme elevations. Available at: https://www.biospace.com/article/surrozen-to-pause-phase-i-crohn-s-trial-over-unfavorable-liver-results/?keywords=surrozen (Accessed November 15, 2022)

[B89] Toyohara T. Roudnicky F. Florido M. H. C. Nakano T. Yu H. Katsuki S. (2020). Patient hiPSCs identify vascular smooth muscle arylacetamide deacetylase as protective against atherosclerosis. Cell Stem Cell 27, 178–180. 10.1016/j.stem.2020.05.013 32619513

[B90] Vittoria Zinzalla B. D.-H. Savchenko A. Rinnenthal J. R. G. Bauer M. Sanderson M. Blake S. (2019). Abstract DDT01-01: BI 905677: a first-in-class LRP5/6 antagonist targeting Wnt-driven proliferation and immune escape. Cancer Res. 79, DDT01–01. 10.1158/1538-7445.AM2019-DDT01-01

[B91] Wang H. Y. Lee K. C. Pei Z. Khan A. Bakshi K. Burns L. H. (2017). PTI-125 binds and reverses an altered conformation of filamin A to reduce Alzheimer's disease pathogenesis. Neurobiol. Aging 55, 99–114. 10.1016/j.neurobiolaging.2017.03.016 28438486

[B92] Wang H. Y. Pei Z. Lee K. C. Lopez-Brignoni E. Nikolov B. Crowley C. A. (2020). PTI-125 reduces biomarkers of alzheimer's disease in patients. J. Prev. Alzheimers Dis. 7, 256–264. 10.14283/jpad.2020.6 32920628

[B93] Wang M. J. Peng X. Y. Lian Z. Q. Zhu H. B. (2019). The cordycepin derivative IMM-H007 improves endothelial dysfunction by suppressing vascular inflammation and promoting AMPK-dependent eNOS activation in high-fat diet-fed ApoE knockout mice. Eur. J. Pharmacol. 852, 167–178. 10.1016/j.ejphar.2019.02.045 30826323

[B94] Wang S. X. Feng Y. N. Feng S. Wu J. M. Zhang M. Xu W. L. (2022b). IMM-H007 attenuates isoprenaline-induced cardiac fibrosis through targeting TGFβ1 signaling pathway. Acta Pharmacol. Sin. 43, 2542–2549. 10.1038/s41401-022-00899-2 35354962 PMC9525664

[B95] Wang J. Xiao Q. Wang L. Wang Y. Wang D. Ding H. (2022a). Role of ABCA1 in cardiovascular disease. J. Pers. Med. 12, 1010. 10.3390/jpm12061010 35743794 PMC9225161

[B96] Wang W. Wang S. Liu X. Gu R. Zhu Y. Zhang P. (2018). Knockdown of ARL4C inhibits osteogenic differentiation of human adipose-derived stem cells through disruption of the Wnt signaling pathway. Biochem. Biophys. Res. Commun. 497, 256–263. 10.1016/j.bbrc.2018.02.066 29432742

[B97] Wanschel A. Guizoni D. M. Lorza-Gil E. Salerno A. G. Paiva A. A. Dorighello G. G. (2021). The presence of cholesteryl ester transfer protein (CETP) in endothelial cells generates vascular oxidative stress and endothelial dysfunction. Biomolecules 11, 69. 10.3390/biom11010069 33430172 PMC7825632

[B98] Wei S. M. Xie C. G. Abe Y. Cai J. T. (2009). ADP-ribosylation factor like 7 (ARL7) interacts with alpha-tubulin and modulates intracellular vesicular transport. Biochem. Biophys. Res. Commun. 384, 352–356. 10.1016/j.bbrc.2009.04.125 19409876

[B99] Wong W. T. Sayed N. Cooke J. P. (2013). Induced pluripotent stem cells: how they will change the practice of cardiovascular medicine. Methodist Debakey Cardiovasc J. 9, 206–209. 10.14797/mdcj-9-4-206 24298311 PMC3846074

[B100] Xie L. Fletcher R. B. Bhatia D. Shah D. Phipps J. Deshmukh S. (2022). Robust colonic epithelial regeneration and amelioration of colitis via FZD-specific activation of Wnt signaling. Cell Mol. Gastroenterol. Hepatol. 14, 435–464. 10.1016/j.jcmgh.2022.05.003 35569814 PMC9305022

[B101] Xie L. Tun C. Liu A. Lu C. Baribault H. Newman M. (2021a). P023 SZN-1326, a Wnt agonist, improved epithelial healing and ameliorated colitis in a chronic DSS model, in Stark contrast to anti-TNF and anti-IL-12/23p40 antibodies. J. Crohn's Colitis 15, S140. 10.1093/ecco-jcc/jjab076.152

[B102] Xie N. Bai Y. Qiao L. Bai Y. Wu J. Li Y. (2021b). ARL4C might serve as a prognostic factor and a novel therapeutic target for gastric cancer: bioinformatics analyses and biological experiments. J. Cell Mol. Med. 25, 4014–4027. 10.1111/jcmm.16366 33724652 PMC8051716

[B103] Yang J. Peng S. Zhang K. (2022). ARL4C depletion suppresses the resistance of ovarian cancer to carboplatin by disrupting cholesterol transport and autophagy *via* notch-RBP-Jκ-H3K4Me3-OSBPL5. Hum. Exp. Toxicol. 41, 9603271221135064. 10.1177/09603271221135064 36366750

[B104] Yin Y. Zeng S. Li Y. Wu Z. Huang D. Gao P. (2020). Macrophage Lxrα reduces atherosclerosis in Ldlr-/- mice independent of Arl7 transactivation. Biochem. Biophys. Res. Commun. 529, 540–547. 10.1016/j.bbrc.2020.06.071 32736671

[B105] Yu X. H. Jiang N. Yao P. B. Zheng X. L. Cayabyab F. S. Tang C. K. (2014). NPC1, intracellular cholesterol trafficking and atherosclerosis. Clin. Chim. Acta 429, 69–75. 10.1016/j.cca.2013.11.026 24296264

[B106] Yuan X. Xiao H. Hu Q. Shen G. Qin X. (2022). RGMa promotes dedifferentiation of vascular smooth muscle cells into a macrophage-like phenotype *in vivo* and *in vitro* . J. Lipid Res. 63, 100276. 10.1016/j.jlr.2022.100276 36089003 PMC9587411

[B107] Yu J. Ming H. Li H. Y. Yu B. Chu M. Zhu H. (2019). IMM-H007, a novel small molecule inhibitor for atherosclerosis, represses endothelium inflammation by regulating the activity of NF-κB and JNK/AP1 signaling. Toxicol. Appl. Pharmacol. 381, 114732. 10.1016/j.taap.2019.114732 31454633

[B108] Zhang D. Qiao X. Yao J. Zhang L. Wu X. Ma J. (2021a). Pronethalol reduces Sox2 (SRY [Sex-Determining region Y]-Box 2) to ameliorate vascular calcification. Arterioscler. Thromb. Vasc. Biol. 41, 931–933. 10.1161/ATVBAHA.120.315571 33297753 PMC8105260

[B109] Zhang L. Huang T. Teaw S. Nguyen L. H. Hsieh L. S. Gong X. (2020). Filamin A inhibition reduces seizure activity in a mouse model of focal cortical malformations. Sci. Transl. Med. 12, eaay0289. 10.1126/scitranslmed.aay0289 32075941 PMC12290962

[B110] Zhang P. Xu Y. Chen S. Wang Z. Zhao L. Chen C. (2022). ARL4C regulates the progression of clear cell renal cell carcinoma by affecting the wnt/*β*-catenin signaling pathway. J. Oncol. 2022, 2724515. 10.1155/2022/2724515 35774359 PMC9239764

[B111] Zhang S. Li L. Wang J. Zhang T. Ye T. Wang S. (2021b). Recent advances in the regulation of ABCA1 and ABCG1 by lncRNAs. Clin. Chim. Acta 516, 100–110. 10.1016/j.cca.2021.01.019 33545111

[B112] Zhao H. P. Jiang H. M. Xiang B. R. (2013). Discontinued drugs in 2012: cardiovascular drugs. Expert Opin. Investig. Drugs 22, 1437–1451. 10.1517/13543784.2013.832198 23992034

[B113] Zhao Z. Song G. Tian H. Yu Y. Tian X. Liu J. (2012). Triacetyl-3-hydroxyphenyladenosine, a derivative of cordycepin, attenuates atherosclerosis in apolipoprotein E-knockout mice. Exp. Biol. Med. (Maywood) 237, 1262–1272. 10.1258/ebm.2012.011401 23239437

[B114] Zhou J. Kang X. An H. Lv Y. Liu X. (2021). The function and pathogenic mechanism of filamin A. Gene 784, 145575. 10.1016/j.gene.2021.145575 33737122

